# *Cenchrus spinifex* Invasion Alters Soil Nitrogen Dynamics and Competition

**DOI:** 10.3390/microorganisms12112120

**Published:** 2024-10-23

**Authors:** Meng Meng, Baihui Ren, Jianxin Yu, Daiyan Li, Haoyan Li, Jiahuan Li, Jiyun Yang, Long Bai, Yulong Feng

**Affiliations:** 1 College of Horticulture, Shenyang Agricultural University, Shenyang 110866, China; 2022220409@stu.syau.edu.cn (M.M.);; 2 Liaoning Key Laboratory for Biological Invasions and Global Changes, College of Bioscience and Biotechnology, Shenyang Agricultural University, Shenyang 110866, China

**Keywords:** plant invasion, interspecific competition, nitrogen addition, nitrogen-cycling microbial communities, soil nitrogen conversion rate

## Abstract

Invasive plants often alter biological soil conditions to increase their own competitiveness. Through indoor simulated nitrogen deposition culture experiments, we investigated the differences in growth indicators and nutrient content levels between the invasive plant *Cenchrus spinifex* Cav. and the native symbiotic plant *Agropyron cristatum* (L.) Gaertn. under diverse nitrogen application modes and planting-competition ratios. Furthermore, we examined the alterations in key microbial communities involved in soil nitrogen cycling of *C. spinifex*. The results indicated that the invasion of *C. spinifex* could inhibit the growth of native plants, and in fact altered the accumulation and transformation processes related to soil nitrogen, resulting in reduced rates of soil nitrogen transformation. The overarching aim of this research was to construct a theoretical foundation for the scientific comprehension of the invasion mechanisms of *C. spinifex*, in order to better prevent the further spread of this invasive plant and mitigate its pernicious impact on the current environment.

## 1. Introduction

Plant invasion is regarded as one of the most salient propelling forces of global ecosystem succession [1]. Biological invasions have profoundly impacted ecosystems by incrementally eroding the latter’s resilience and attenuating species richness. Invasive plants commonly manifest heightened competitiveness, which can be further potentiated via mechanisms such as the rhizosphere secretion of specific allelopathic substances [2]. *Cenchrus spinifex*, belonging to the Poaceae family and the Cenchrus genus, is an annual plant that has gained notoriety as one of the most invasive malignant weeds worldwide [3]. The origin of *C. spinifex* was in sandy soil in North America and tropical coastal areas. In recent years, *C. spinifex* has invaded northeast China on a large scale; it is characterized by an extensive range, significant damage area, and rapid transmission speed [4]. The extant invasion status is acutely grave and demands expeditious prophylactic and governance measures [5]. Over time, it will profoundly affect the development of agriculture and animal husbandry, resulting in economic losses and posing a threat to ecosystem security [4]. Therefore, the urgent investigation of the invasive species *C. spinifex* constitutes a crucial research focus for both scientific inquiry and practical applications.

Since the advent of the Industrial Revolution, there has been considerable augmentation in the levels of nitrogen emissions and agricultural nitrogen fertilizer utilization, giving rise to intensified atmospheric nitrogen deposition and a considerable upsurge in available nitrogen content in the soil. This has engendered alterations in soil’s physical and chemical attributes, thereby instigating a succession of ecological and environmental predicaments [6]. The alteration of the nitrogen pool by invasive plants encompasses multiple pathways and characteristics, exerting significant influences on ecosystems; these influences primarily involve nitrogen uptake and conversion in the soil, interactions with microorganisms, and nitrogen metabolism and allocation within plants [7]. During the invasion process, invasive plants demonstrate marked disparities, compared to native plants, in nutrient absorption, substance regulation, and secretion. Invasive plants can alter the quantities and distribution ratios of soil nutrient forms, especially nitrogen, which is inextricably associated with plant growth. Changes in nutrient content and form can affect the survival and competitive ability of local plants, thereby altering the species composition and community structure of the ecosystem [8]. *Solidago canadensis* L. (indigenous to North America, this C4 perennial herbaceous plant is classified within the genus Solidago of the Asteraceae family) possesses a well-developed root system, facilitating the rapid uptake of ammonium and nitrate nitrogen from soil, compared to native plants, during the early growth stage. Additionally, under conditions of nitrogen surplus, *S canadensis* L. can convert the excess nitrogen into organic nitrogen for storage, allowing subsequent utilization during later growth stages or periods of nitrogen scarcity [8]. Recent research has predominantly examined alterations in “total soil” and inorganic nitrogen resulting from plant invasion. The invasion of *S canadensis* L., for instance, has increased the soil total nitrogen content (24.7%) but decreased the ammonia (12.5%) and nitrate nitrogen (10.8%) content levels [9]. Investigation of *Mikania micrantha* Kunth (indigenous to South and Central America, this C3 perennial herbaceous plant or shrub-like climbing vine belongs to the genus Pseuderanthemum within the Asteraceae family) revealed no statistically significant systematic variation in the soil alkaline nitrogen content between pre- and post-invasion levels [10]. In an in-depth exploration of *Lantana camara* L. (indigenous to the tropical and subtropical regions of the Americas, this perennial shrub belongs to the genus Lantana and is classified within the Verbenaceae family) within deciduous forest biomes, Sharma et al. observed that, with the intensification of invasion, the amount of detritus incrementally increased. The elevated nitrogen and diminished lignin contents in litters of *Lantana camara* L. markedly enhanced both the availability of soil nitrogen (17.9%) and the total nitrogen content (23.5%) [11]. Furthermore, when *Spartina alterniflora* Loisel. invaded an area previously dominated by *Phragmites communis* (a native plant in the Yangtze River estuary), the *S. alterniflora* Loisel. community with N supplementation exhibited the maximal soil N-fixation rate (47.6%) and *nifH* gene abundance (38.3%) across all experimental treatments [12]. These research outcomes accentuate the need for prolonged, comprehensive experimental inquiries concentrating on key invasive plant taxa. Moreover, it can be inferred that the preferential absorption of disparate nitrogen forms by the subterranean root biomass of invasive plants, along with the composition of chemical substances within the litter, could potentially account for the modifications in soil nitrogen dynamics and other edaphic attributes found in invasion-prone regions [13].

Recent investigations have disclosed that soil microorganisms are pivotal in modulating plant competitiveness. For example, rhizosphere microbial consortia may disparately impact the soil nutrient bioaccessibility relative to diverse plant taxa, thereby influencing the latter’s competitive equilibria [14]. Following the incursion of *Centaurea cyanus* DC (Asteraceae), an annual or biennial C3 herbaceous plant indigenous to Europe, the species contributed substrates to the soil via litter decomposition and root exudation, thereby modifying the composition and functionality of microbial assemblages [15]. Soil microorganisms constitute indispensable elements within soil ecosystems, exerting a crucial influence on nutrient cycling and regulating plant growth processes [16]. The structure of soil microbial communities might undergo changes due to plant invasion, subsequently exerting a considerable influence on the plants’ invasive potential [17]. *Phragmites communis* (a C3 perennial herbaceous plant classified within the genus Phragmites of the Poaceae family) exhibits a significantly higher litter decomposition rate contrasted with native plant species, thereby inducing alterations in soil conditions that might potentially influence the functionality of soil microbial communities [18,19]. The interaction between invasive plants and soil microorganisms significantly affects the adaptability and competitiveness of invasive species, playing a critical role in these species’ successful invasions. During the summer, the relative abundance of Desulfobulbaceae associated with the *Spartina alterniflora* Loisel. (a C3 perennial herbaceous plant classified within the genus Spartina of the Poaceae family) invasion is significantly greater than that observed in other vegetation types, reaching 3.15% [20]. *Ageratina adenophora* (indigenous to South America, a perennial C3 herbaceous plant classified within the Ageratina genus of the Asteraceae family) manifests a preferential accumulation of certain microorganisms within the rhizosphere soil milieu, particularly Sphingomonas and Steroidobacter, while concurrently attenuating the abundance of Gaiella and Gp6. This distinctive microbial community structure may potentiate the invasive plant’s aptitude for adaptation and competition in novel ecological niches [21]. Modifications within soil microbial communities forge a mechanistic connection between invasive plants and alterations in ecosystem nutrient cycling. Nevertheless, factors influencing the growth feedback mechanism exerted by invasive plants on dominant local species remain ambiguous, necessitating further investigation into the effects of invasive plants on soil microorganisms and interspecific competition.

Soil nitrogen-cycling microbial communities are crucial for ecosystem nitrogen cycling, and invasive plants may impact this process by manipulating the community structure and diversity of key soil microorganisms involved in nitrogen transformation [22]. Families such as Hyphomonadaceae (17.5%) and Nitrospirae (3.5%) in the order Rhodobacteriales exhibit higher abundance levels within the rhizosphere soil associated with invasive plants. Additionally, nitrogen-fixing bacteria (21.2%) involved in nitrogen cycling also demonstrate increased abundance during the invasion process [23]. The impact of *Flaveria bidentis* (an annual C4 herbaceous plant affiliated with the Asteraceae family) invasion on soil microbial communities resembles the observed effects of nitrogen availability, typically leading to a rise in both the profusion (27%) and metabolic activity (34.1%) of ammonia-oxidizing archaea. This enhancement of soil fecundity subsequently engenders a positive feedback mechanism that facilitates the growth of invasive plants, thereby exacerbating the dynamics of invasion. [24]. Moreover, the abundance of ammonia-oxidizing bacteria in invaded soil can increase by 37.4%, alongside a 23.6% rise in the total nitrification rate, providing sufficient nitrogen to support the continued invasion of invasive plants [25]. Invasive plants can deplete the abundance of ammonia-oxidizing bacterial communities by 18.9% and consequently influence nitrogen cycling processes [22]. Invasive plants can also modify the microbial community structure of denitrifying bacteria, thereby reducing their denitrification capacity and resulting in reduced concentrations of nitrate nitrogen (34.1%) and ammonia oxides (23.7%) [26,27]. However, more in-depth research is required to illuminate the influence of invasive plants on the nitrogen-transformation functionalities of microorganisms.

The present study investigated the effects of *C. spinifex* invasion on soil nitrogen cycling characteristics and four key soil nitrogen-cycling microbial communities, including nitrogen-fixing bacteria, ammonia-oxidizing bacteria (AOB), ammonia-oxidizing archaea (AOA), and denitrifying microorganisms. This was accomplished via field and pot experiments to determine the competitive mechanism through which this invasive species surpasses the symbiotic native perennial herbaceous plant of the Poaceae family, *Agropyron cristatum*, within the context of nitrogen deposition. We quantified the variations in plant performance, the nitrogen uptake in diverse plant tissues of both species, and the abundance of key microbial communities involved in soil nitrogen transformation. Our objective was to discern the primary contributors among the key nitrogen-cycling microbial communities that enhance soil available nitrogen to *C. spinifex* and to assess how these factors affect this species’ competitiveness with native plants. We assumed the following: (1) the competitive responses may vary between alien invasive plants and native plants, with soil nitrogen availability mediating the competitive dynamics involving invasive plants; (2) nitrogen supplementation could positively influence the growth levels and nitrogen utilization strategies of invasive plants; and (3) invasion by *C. spinifex* would likely alter the soil environment, actively recruiting microorganisms involved in soil nitrogen cycling, and thereby influence the overall process of soil nitrogen cycling.

## 2. Methods and Materials

### 2.1. Description of the Study Area

This study was conducted on the southern edge of Horqin Sandy Land, located in Liaoning Province, Zhangwu County, northern China (122°33′ E, 42°48′ N). The study area is characterized by a temperate monsoon climate with an annual precipitation ranging from 450 to 550 mm, predominantly occurring in July and August. The average annual relative humidity is between 60% and 65%. The selected site exhibits minimal human and livestock disturbance and has a low level of nutrient content and poor physical properties (Supplementary File/Table S1). Dominant native plant species include *Agropyronum caucasicum*, *Setaria viridis* (a C4 annual grass classified within the Agrostis genus of the Poaceae family), *Lespedeza davurica* (a C3 shrub classified within the leguminous genus Lespedeza), and *Digitaria sanguinalis* (a C4 annual grass classified within the Agrostis genus of the Poaceae family). As a species well-adapted for sandy grassland conditions, *Cenchrus spinifex* has become widely distributed in this area, leading to significant transitions into pure *C. spinifex* invasion habitats. Consequently, four distinct habitat types reflecting varying degrees of invasion were identified: bare ground habitat (LD; devoid of vegetation); native plant community habitat (BY; where native plants cover > 90%); mixed habitat, comprising both *C. spinifex* and native plants (HD; exhibiting similar coverage between the two groups); and the invasive plant monodominant community sample habitat (SY; where *C. spinifex* cover > 90%) (Supplementary File/Figure S1).

### 2.2. Field Sampling

In September 2021, we delineated a rectangular area measuring 100 × 300 m within the sandy grassland of Horqin that encompassed these four habitats with differing levels of invasion. Within each invasive habitat type, we established four plots, each measuring 1 × 1 m, ensuring a minimum spacing of over 5 m between them. Within each plot, the rhizosphere soil of the plant was collected using a five-point sampling method, which was followed by the removal of roots and gravel using a 2 mm sieve, before storage of the samples in Ziplock bags. The samples were then placed in an ice box and transported to the laboratory, where they were divided into two portions; one underwent physical and chemical analysis after air drying and sieving through a 0.25 mm mesh, while the other was immediately stored in a freezer for subsequent DNA extraction.

### 2.3. Potting Experiment Design

The pot culture experiment was conducted at the Baicaoyuan Teaching and Research Base of Shenyang Agricultural University (41°50′ N, 123°34′ E). The experimental base was located in a temperate semi-humid continental monsoon climate, with an average temperature of 26 °C and a relative humidity of 80% rH recorded inside the greenhouse. The average temperature outside the greenhouse was 24 °C, with a relative humidity of 86% rH. The cold shed adopted a natural light cycle.

The soil for the potted plants was obtained from a 10 cm soil layer taken from the Horqin sandy grassland. Prior to potting, it underwent sieving through a 10 mm sieve to remove organic residues, large soil animals, and large stone fragments, ensuring a uniform mixing of the soil. Small pots designated for individual planting contained 1.25 kg of soil per pot, while larger pots for mixed planting were filled with 5 kg of soil per pot. Seeds of the plant *C. spinifex* and the native plant *A*. *cristatum* were collected from the research area and subjected to germination treatment before seedlings were cultivated. Once the plants reached three pairs of leaves, seedlings exhibiting consistent growth were selected for each species. Subsequently, thinning, supplementing, and weeding took place (retaining well-growing plants—one plant per pot for single planting, four plants per pot for mixed planting according to the agreed planting ratio, and equal spacing between plants during final seedling setting in mixed planting) until fertilization treatment commenced.

The experiment focused on *C. spinifex* (C) and its native symbiotic plant, *A. cristatum* (A), as research subjects, and was divided between single- and mixed-species planting. Each plant (C and A) was replicated eight times per plant for single-species planting. For mixed planting, three different planting ratios (1:3, 2:2, and 3:1, with different competing objects and densities) were used along with three planting modes (1C + 3A, 2C + 2A, and 3C + 1A), and each was replicated eight times. The nitrogen fertilizer treatments included five conditions: high and low concentrations of ammonium nitrogen (NH_4_Cl) (nitrogen content of 400 and 200 mg), high and low concentrations of nitrate nitrogen (Ca(NO_3_)_2_·4H_2_O) (nitrogen content of 400 and 200 mg), and a control group without added nitrogen. In the experiment, nitrogen fertilizer was applied in solution form over eight weeks from the first fertilization (Supplementary File/Figure S2).

### 2.4. Experimental Sampling

After a thorough rinsing of the roots with tap water, the plants were divided into two components: aboveground biomass and underground biomass. They were subsequently subjected to thermal treatment at 105 °C for 2 h, followed by drying at 65 °C for 48 h, to determine their dry weight. Plant height was measured using a ruler. Soil samples surrounding the plant roots were collected using the shaking-soil method. The root soil was placed in sterilized polyethylene bags, promptly transferred to an ice box, and brought back to the laboratory. A portion of the soil was extracted and stored at a low temperature of 4 °C to analyze its fundamental physical and chemical properties. The remaining portion was stored in an −80 °C freezer for future microbiological examination.

### 2.5. Soil Physicochemical Analysis

The total soil nitrogen (TN) was ascertained using the Kjeldahl method. The soil pH and electrical conductivity (EC) were determined by employing the glass-electrode approach at a soil-to-water mass ratio of 1:2.5. The concentrations of ammonium (NH_4_^+^-N) and nitrate (NO_3_^−^-N) were gauged via potassium chloride solution extraction-flow analysis with a QC8000 analyzer. The total organic carbon content of the soil (SOC) was appraised by external heating with potassium dichromate. Total phosphorus in the soil (TP) was determined using molybdenum–antimony resistance colorimetry, while available phosphorus (AP) was extracted through sodium bicarbonate molybdenum–antimony resistance colorimetry. Indoor incubation methods were used to measure soil nitrogen mineralization. Specifically, 30 g of the screened air-dried soil specimens were weighed and placed in 250 mL plastic wide-mouthed flasks. They were then adjusted to a 20% mass water content with deionized water before being pre-incubated for seven days at 25 °C in an incubator wrapped with polyethylene film that had two small holes in it. Subsequently, they were partitioned into three cohorts after pre-incubation: one group received 50 mg·kg^−1^ of ammonium sulfate, another group received 50 mg·kg^−1^ of sodium nitrate solution, and the third group was prepared as a control. The humidity levels for all three groups were adjusted to 40% of their respective soils’ water-holding capacities before being resealed and further incubated in the dark at 25 °C for an additional fourteen days. Variations in the soil moisture content were monitored by weighing and replenishing the soil moisture with deionized water as required. On both day zero and day fourteen of incubation, 75 mL of a 2M KCl solution was added; the specimens then underwent agitation in a constant-temperature oscillating apparatus at 25 °C for 1 h. The resultant filtrate was analyzed using the Auto Analyzer 3 System (SEAL Analytical Ltd., Southampton, UK) to quantify the concentrations of nitrate and ammonium nitrogen within the soil [28].

### 2.6. Plant Processing

After pulverizing the plants with a powdered prototype, the Smartcherm140 automatic intermittent chemical analyzer was utilized to determine the levels of total nitrogen and total phosphorus.

We calculated the relative interaction index (RII) using the formula RII = (Bw Bo)/(Bw + Bo) [29], where Bw represents the biomass of plants under mixed planting and Bo represents the biomass of plants under single planting. When 0 < RII ≤ 1, it indicates a promoting effect; when −1 < RII ≤ 0, a significant competitive interaction effect is present, manifested as an inhibitory effect; when RII = 0, the promoting and inhibitory effects cancel out and reach a balance [30]. The RII value can be effectively used to compare competition between target plants and native plant species [31].

### 2.7. Microbial Analysis

Soil microbial DNA was extracted from approximately 0.25 g of soil from each sample, employing the Fast DNA SPIN extraction kit (MP Biomedicals, Santa Ana, CA, USA), and subsequently preserved at −20 °C until further analysis. The quantity and quality of extracted DNA were evaluated using a NanoDrop ND-1000 spectrophotometer (Thermo Fisher Scientific, Waltham, MA, USA). Quantitative fluorescence PCR was used to determine the abundance of soil microorganisms. PCR amplification of all genes (N-fixing bacteria (*nifH*) for nitrogen fixation and the ammonia-oxidizing archaea (AOA) and ammonia-oxidizing bacteria (AOB), together with ammonia monooxygenase genes (*amoA*) and denitrifying bacteria (*nirK*) associated with nitrite reduction [32]) was performed, using the previously described primers (Supplementary File/Table S2). The amplification reaction system had a total volume of 20 μL, consisting of 10 μL of 2 × GoTaq^®^ qPCR Master Mix, 0.5 μL each of the upstream and downstream primers at a concentration of 10 μmol/L, 2 μL of the DNA template (1–10 ng), and 7 μL of sterilized ultra-pure water. The enhanced 96-PCR plate was subjected to quantitative fluorescence PCR, with three replicates per sample. The amplification conditions included predenaturation at 95 °C for 30 s, denaturation at 95 °C for 5 s, annealing at 60 °C for 40 s, and extension at 72 °C for 30 s, with 40 cycles. Quantification was based on the increased fluorescence intensity of the SYBR Green dye during amplification. Standard curves were established through serial dilutions of linearized plasmids encompassing the genes under scrutiny. Two to three no-template controls were incorporated for each quantitative PCR assay, and these controls yielded negligible or null values. The cycle number (Ct value) at which the fluorescence intensity reached the threshold was used to construct the standard curve. The Ct values of the obtained samples were substituted into the standard curve to calculate the abundance of the corresponding functional genes for each sample.

The relevant formula was as follows:

$$\mathrm{copies}=10^{\frac{\mathrm{Ct}-b}{k}}$$

For each concentration of the standard substance, quantitative real-time polymerase chain reaction (qPCR) assays were carried out to obtain the corresponding Ct values. A standard curve was constructed by plotting the logarithm of the copy number of the standards on the x-axis against their respective Ct values on the y-axis. Subsequently, the copy numbers of the unknown samples were ascertained based on this standard curve. The qPCR reactions for the unknown samples yielded their Ct values, which were then substituted into the equation derived from the standard curve to calculate the copy number of the target nucleic acid in these samples. In this equation, *k* represents the slope and b denotes the intercept.

### 2.8. Soil N-Cycling Rates

The soil nitrogen mineralization was determined using an in vitro incubation technique. A total of 30 g of each sieved and air-dried soil sample was transferred into a 250 mL conical flask, where the moisture content was adjusted to 20% with deionized water. Subsequently, the flasks were sealed with a polyethylene film which had two small perforations for gas exchange and incubated at 25 °C for a pre-incubation period of seven days. Following this pre-incubation phase, the samples were divided into three groups: one group received ammonium sulfate at a concentration of 50 mg·kg^−1^, another group was treated with a solution of calcium nitrate tetrahydrate, and the third group served as a control. The moisture levels across all samples were standardized to calculate 40% of their respective water-holding capacities. All flasks were resealed and subjected to dark incubation at 25 °C for an additional fourteen days. Changes in soil moisture were monitored through weighing, with deionized water added as necessary to maintain consistent moisture levels throughout the experiment. On both day zero and day fourteen post-incubation, each flask received an additional 75 mL of 2 M KCl; these mixtures were agitated for one hour in a constant-temperature shaker set at 25 °C. Finally, the concentrations of nitrate and ammonium in the filtrate were quantified using a continuous flow analyzer (SEAL Analytical, Norderstedt, Germany).

The soil nitrogen conversion rate was calculated using the following equations:

$$\mathrm{Net}\mathrm{mineralization}\mathrm{rate}\;(NMR)=\frac{\left(M_{t}+N_{t})-(M_{0}+N_{0}\right)}{t}$$

$$\mathrm{Net}\mathrm{nitrification}\mathrm{rate}\;(NNR)=\frac{N_{t}-N_{0}}{t}$$

$$\mathrm{Net}\mathrm{ammonification}\mathrm{rate}\;(NAR)=\frac{M_{t}-M_{0}}{t}$$

The rates were expressed in mg·g^−1^·d^−1^. *N*_0_ and *N_t_* represent the initial and final concentrations of nitrate nitrogen, while *M*_0_ and *M_t_* refer to the initial and final concentrations of ammonium nitrogen [33].

### 2.9. Statistical Analysis of the Data

The data from this experiment were subjected to statistical analysis, calculations, and charting using Microsoft Office Excel 2013 (Microsoft, Redmond, DC, USA). Analysis of variance (ANOVA) on the experimental data was performed using SPSS 23.0 (IBM, Armonk, NY, USA), and when significant differences were found, multiple comparisons were conducted using the Duncan method with a significance level of 0.05. A three-factor analysis of variance was employed to analyze the effects of different forms of nitrogen fertilizer (NO_3_^−^-N and NH_4_^+^-N) and nitrogen fertilizer concentration (high and low), as well as different planting ratios of single and mixed species (1C + 3A, 2C + 2A, and 3C + 1A), on the plant height, aboveground biomass, underground biomass, and total biomass of the two plants. Additionally, a two-factor analysis of variance was used to investigate the impacts of different periods and habitats on the physicochemical properties of the surface and rhizosphere soil of *C. spinifex*. Network heatmaps depicting environmental factors and soil nitrogen cycling microbial-community abundance were generated using the OmicShare tool (https://www.omicshare.com/tools/index.php, accessed on 3 February 2023).

We utilized AMOS7.0 software (IBM, Armonk, NY, USA) to conduct structural equation modeling to investigate the primary pathways that might elucidate the accelerated production of available nitrogen in both the field and potted experiments involving *C. spinifex*. Initially, we constructed a prior model based on general ecological knowledge to establish causal relationships from our observed data. Subsequently, we calibrated the model using gene abundance data, the nitrogen conversion rate, and soil physicochemical properties, aiming to quantify the impact of *C. spinifex* invasion on soil nitrogen conversion processes. The initial assumption was supported by its alignment with existing data. In cases where the initial model was rejected due to inadequate data fitting, alternative models were considered until satisfactory support was achieved. Ideally, subsequent research should confirm the inference made by the revised model. Finally, we employed the v2 test, Bentler Bonnet normalization fit index, comparative fit index (CFI), and goodness of fit index (GFI) as evaluation metrics for the scanning electron microscopy’s goodness-of-fit assessment. The insignificant difference observed in the v2 test (*p* > 0.05) provided evidence for the optimal fitness of the selected SEM [34].

## 3. Results

### 3.1. The Impact of Invasion of Cenchrus spinifex on the Abundance of Key Microbial Communities in Soil Nitrogen Cycling

#### 3.1.1. The Impact of Invasion on Soil Physicochemical Properties and Nitrogen Conversion Rate

The TN content in the soil colonized by *C. spinifex* in HD was significantly higher than that in BY (*p* < 0.05) (Table 1). The soil AN content of SY was significantly higher than that of HD (*p* < 0.05). As the extent of invasion increased, the soil TP, SOC content, and EC manifested substantial decrements (*p* < 0.05). Moreover, the soil AP content in SY was markedly lower than that in BY (*p* < 0.05), while the soil NNR in HD was significantly increased in comparison to LD (*p* < 0.05).

#### 3.1.2. The Impact of Invasion on the Abundance of Key Microorganisms Involved in Soil Nitrogen Transformation

Employing qPCR assays, the levels of the *nifH*, AOA-*amoA*, AOB-*amoA*, and *nirK* genes were quantified in the soil specimens from four habitats characterized by distinct degrees of *C. spinifex* invasion. The amplification efficacies of the standard curves surpassed 93% (Supplementary File/Figure S4), the correlations exceeded 0.99 (Supplementary File/Figure S3), and all the melting curves manifested as single peaks (Supplementary File/Figure S5), substantiating the validity of the curve-analysis outcomes.

**Figure 1:**
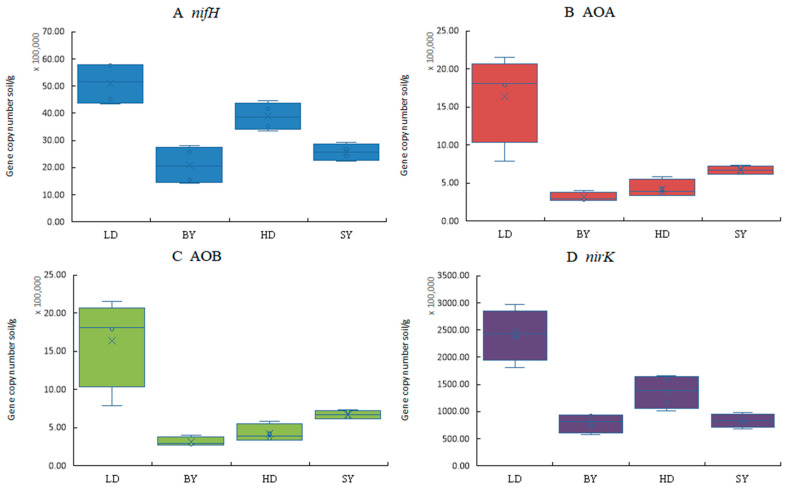
Abundance of key microorganisms associated with nitrogen transformation in different habitats invaded by *C. spinifex* ((**A**) nitrogen-fixing gene *nifH*, (**B**) ammonia-oxidizing archaea gene AOA, (**C**) ammonia-oxidizing bacteria AOB, and (**D**) denitrifying bacteria *nirK*). LD refers to bare land, BY refers to the sample land of the native plant community, HD refers to the sample land of mixed communities of *C. spinifex* and native plants, and SY refers to the invasion sample land, composed of patches inlaid by monodominant communities of *C. spinifex* (*n* = 3).

In contrast to LD, the abundance of the soil nitrogen-fixing gene *nifH* showed a significant decrease (*p* < 0.05) with increasing levels of *C. spinifex* invasion (Figure 1). Similarly, the abundance of AOA-*amoA* significantly decreased with the invasion of *C. spinifex*, compared to LD (*p* < 0.05). The abundance of AOB-*amoA* in soils invaded by *C. spinifex* was significantly higher than in other habitats (*p* < 0.05). Compared to LD, the abundance of *nirK* showed a marked reduction with increasing levels of invasion (*p* < 0.05).

#### 3.1.3. Heat Map Analysis of the Correlation Between Soil Environmental Factors and the Abundance of Nitrogen-Transformation Functional Genes

**Figure 2:**
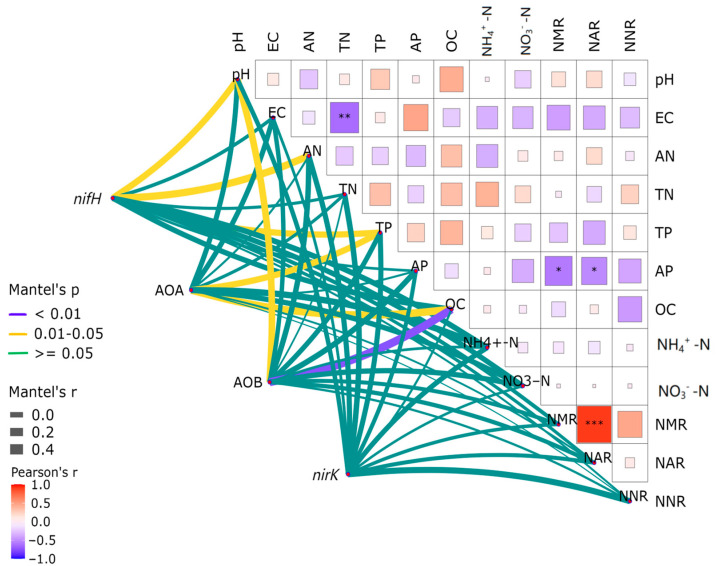
Correlation heat map of nitrogen-transformation function gene abundance and physical and chemical properties of *C. spinifex* invading different habitats at different stages. The right triangle diagram shows Pearson correlation coefficients associated with the physical and chemical properties of different soils and nitrogen conversion rates. See the left line diagram for Mantel test results. For Mantel analysis, we used proportional values of soil physicochemical properties and nitrogen conversion rates. Before calculating the Euclidean distance, the alpha diversity was scaled. The meanings of abbreviations for soil physical and chemical properties, functional genes, and nitrogen conversion rate are shown in the text. The line width corresponds to the partial Mantel r statistic, and the line color represents statistical significance based on 999 permutations. The environmental factors were compared in pairs. Color gradients are used to express Pearson correlation coefficients. “*” indicates a significant correlation (*p* < 0.05); “**” represents a very significant association (*p* < 0.01); “***” represents a very significant association (*p* < 0.001), and the number represents an F value. (*n* = 3).

The abundance of *nifH* demonstrated pronounced correlations with the soil pH, AN, and TP contents (*p* < 0.05), while the abundance of AOA-*amoA* showed significant associations with the soil TP and SOC contents (*p* < 0.05) (Figure 2). A salient relationship was discerned between *amoA* abundance and both the soil pH and SOC contents (*p* < 0.05), while the abundance of *nirK* presented no significant correlation with any of the soil’s physicochemical properties (*p* > 0.05). According to Pearson’s correlation analysis, there was a highly significant negative correlation between NMR and NAR.

### 3.2. The Effects of Different Forms of Nitrogen Addition on the Abundance of Rhizosphere Soil Microbial Communities of the Invasive Plant C. spinifex and Native Symbiotic Plants

#### 3.2.1. Plant Height

Plant type, planting mode, and nitrogen-addition level all exerted a pronounced effect on plant height (*p* < 0.01). No appreciable disparity in plant height was discerned under the interaction between plant type and planting mode, but significant variances were detected (*p* < 0.01) for the interactions encompassing plant type and nitrogen-addition level, between planting mode and nitrogen addition, and across the combined elements of plant type, planting mode, and nitrogen addition (Supplementary File/Table S3).

When the nitrogen-addition level was homogeneous for *A. cristatum*, the planting mode significantly impacted plant height (*p* < 0.05). On the contrary, when the planting mode was the same for *A. cristatum*, dissimilar nitrogen-addition treatments under the mixed planting mode significantly influenced plant height (*p* < 0.05) (Supplementary File/Figure S6A). The outcomes demonstrated that the height of *A. cristatum* plants in monoculture was significantly superior to that in polyculture contexts. Under mixed cropping conditions, when high concentrations of ammonium nitrogen were added, the overall height of *A. cristatum* plants was significantly higher than values observed under other nitrogen-addition levels.

When the nitrogen-addition level was the same for *C. spinifex*, marked disparities in plant height were discerned among the different planting modes (*p* < 0.05) (Supplementary File/Figure S6B). In contrast, when the nitrogen-addition levels varied while the planting mode remained unchanged, significant differences in plant height were observed (*p* < 0.05). The results indicated that the height of *C. spinifex* plants in single-species samples was significantly higher than that in mixed-species samples. Notably, upon the addition of high concentrations of ammonium nitrogen, the height of *C. spinifex* plants was significantly lower, compared to those under other nitrogen-addition levels.

#### 3.2.2. Plant Biomass

Plant species, planting mode, nitrogen-addition levels, and their interactions all exerted a pronounced and substantive effect on total biomass (*p* < 0.01) (Supplementary File/Table S3) (Supplementary File/Figure S7A). When the nitrogen-addition level was uniform for *A. cristatum*, the planting mode had a significant impact on the total biomass of the plant (*p* < 0.05). On the contrary, when the nitrogen-addition levels varied while the planting patterns remained the same, there was a significant difference in the total biomass of *C. spinifex* under single planting and the 2A + 2A, 1C + 3A, and single-species *A. cristatum* planting patterns (*p* < 0.05). The results demonstrated that the total biomass of *A. cristatum* in single species was significantly higher than that in mixed species. Moreover, the influence of nitrogen-addition treatments on the total biomass of *A. cristatum* demonstrated variability among the different planting modes.

*C. spinifex* manifested marked disparities under identical planting patterns and diverse nitrogen levels (*p* < 0.05) (Supplementary File/Figure S7B). The outcomes signified that the total biomass of the single-species *C. spinifex* sample was significantly lower than those of mixed species (*p* < 0.05). In particular, when low concentrations of nitrate nitrogen were administered, the total biomass of *C. spinifex* was significantly higher than at other nitrogen-addition levels (*p* < 0.05).

#### 3.2.3. Interaction Index

**Figure 3:**
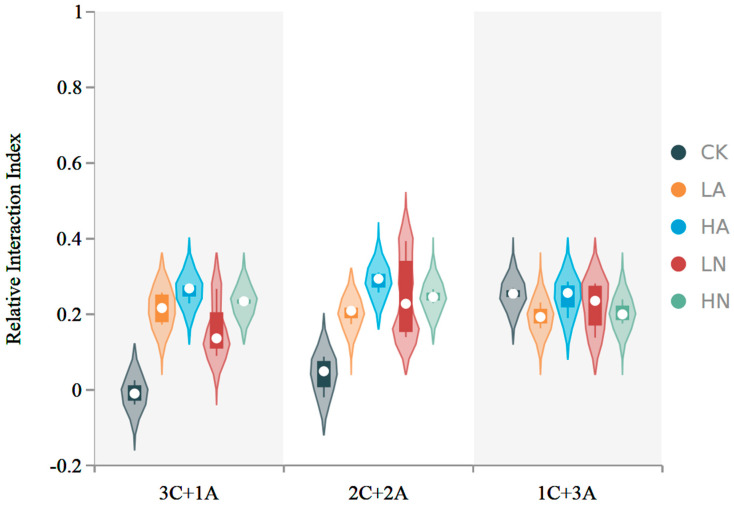
The relative interaction index of the plants across three cultivation paradigms and under five nitrogen-addition scenarios. 3C + 1A: *C. spinifex* and *A. cristatum* in a 3:1 ratio, 2C + 2A: *C. spinifex* and *A. cristatum* in a 2:2 ratio, 1C + 3A: *C. spinifex* and *A. cristatum* in a 1:3 ratio. CK: control, LA: low-concentration ammonium nitrogen, HA: high-concentration ammonium nitrogen, LN: low-concentration nitrate nitrogen, HN: high-concentration nitrate nitrogen (*n* = 3).

The *RII* value of *C. spinifex* and *A. cristatum* exceeded 0, signifying an enhanced nitrogen utilization rate when contrasted to the native plant *A. cristatum*. The encroachment of *C. spinifex* significantly reduced the *RII* values, indicating that this invasion suppressed the growth of *A. cristatum* (Figure 3).

#### 3.2.4. Response of the Rhizosphere Soil Environment to the Addition of Nitrogen and Interspecific Competition

The *C. spinifex* was not cultivated under homogeneous nitrogen-addition levels, and this gave rise to pronounced discrepancies in soil total nitrogen content (*p* < 0.05) (Supplementary File/Figure S8A). At the same nitrogen-addition level, considerable variations in the total nitrogen content were discerned within the soil (*p* < 0.05). The results showed that the rhizosphere soil presented a significantly elevated total nitrogen content in contrast to monocultural circumstances. In single-species samples, the total nitrogen content in the rhizosphere soil was significantly higher than the values found for other nitrogen-addition levels. In mixed-species cases, the total nitrogen content of the soil supplemented with a high concentration of ammonia nitrogen was strikingly higher than that affiliated with other nitrogen-addition levels.

The total phosphorus content in the rhizosphere showed significant differences (*p* < 0.05), as did the levels of nitrogen added (*p* < 0.05) (Supplementary File/Figure S8B). The findings demonstrated that the total phosphorus content was significantly higher than the values associated with several other nitrogen species.

The pH within the rhizosphere under elevated nitrogen-addition conditions showed significant differences (*p* < 0.05) (Supplementary File/Figure S8C). At different levels of nitrogen addition for the same planting pattern, there were significant differences in the soil pH (*p* < 0.05). The outcomes displayed significant differences in soil pH within the rhizosphere. Specifically, the rhizosphere soil pH declined upon ammonia supplementation and increased with the addition of nitrate.

Excluding the control treatment, substantial discrepancies in the electrical conductivity of the rhizosphere soil were discerned among the nitrogen-addition treatments (*p* < 0.05) (Supplementary File/Figure S8D). There were significant differences in the electrical conductivity of the rhizosphere soil of *C. spinifex* under the same planting mode and different nitrogen-addition levels (*p* < 0.05). The results showed that when nitrogen was not administered or low concentrations of nitrate nitrogen were added, the electrical conductivity of the rhizosphere soil of *C. spinifex* was significantly lower than found in the other three nitrogen-addition levels. On the contrary, with both high and low concentrations of ammonia nitrogen and high concentrations of nitrate, the electrical conductivity of the rhizosphere soil of *C. spinifex* was significantly higher than values determined under the other treatments. In mixed planting situations, when the nitrogen addition was the same, the soil conductivity generally declined as the number of *C. spinifex* plants increased.

There was a significant difference in the organic carbon content of the rhizosphere soil when low concentrations of ammonia and nitrate and a high concentration of ammonia were added (*p* < 0.05) (Supplementary File/Figure S8E). There was a significant difference in the organic carbon content of the rhizosphere soil of *C. spinifex* under the same planting mode but different nitrogen-addition levels (*p* < 0.05). The results indicated that the organic carbon content in the rhizosphere soil of *C. spinifex* under single planting conditions was higher than values recorded under mixed planting conditions. Adding ammonium and nitrate reduced the organic carbon content of the rhizosphere soil.

In planting modes with similar nitrogen-addition levels, pronounced disparities in the ammonia content were discerned in the rhizosphere soil between the control group and the treatments with low concentrations of ammonium and nitrate (*p* < 0.05) (Supplementary File/Figure S8F). Additionally, under varying nitrogen-addition levels, significant differences were detected in the ammonia content of the rhizosphere soil associated with *C. spinifex* (*p* < 0.05). The outcomes indicate that the ammonium content values in mixed planting scenarios were significantly higher than those in the single-planting cases. Moreover, when high levels of ammonium were administered, the ammonium content in the rhizosphere soil of *C. spinifex* underwent a marked increase.

Significant differences were observed in the nitrate nitrogen content in rhizosphere soil (*p* < 0.05) (Supplementary File/Figure S8G). Additionally, notable variations in the nitrate nitrogen content of the rhizosphere soil were found for the same planting mode and different nitrogen-addition levels (*p* < 0.05). The findings indicate that the addition of high concentrations of nitrate significantly elevated the nitrate content in the rhizosphere soil of *C. spinifex* compared to other nitrogen-addition levels. In the mixed planting scenario, the nitrate nitrogen content in the rhizosphere soil of *C. spinifex* significantly decreased with the increase in the number of *C. spinifex* plants.

#### 3.2.5. Response of the Nitrogen Conversion Rate in Rhizosphere Soil to the Addition of Nitrogen and Interspecific Competition

**Figure 4:**
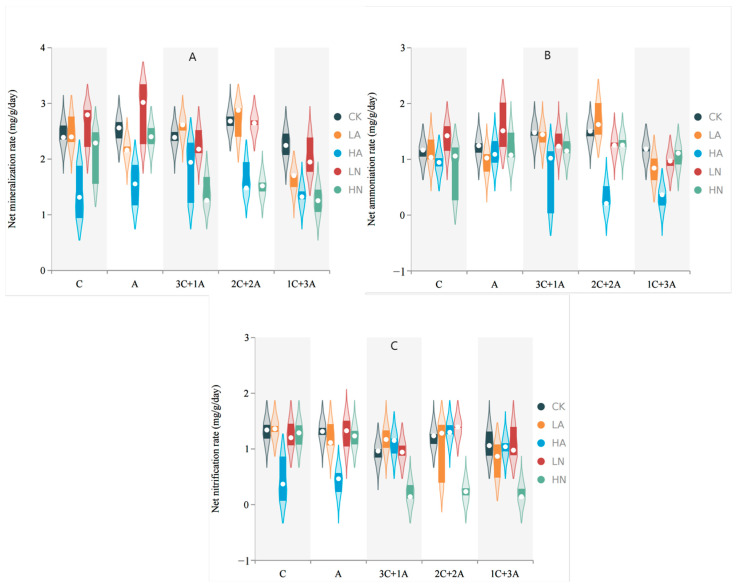
Nitrogen conversion rate in rhizosphere soil ((**A**) net mineralization rate, (**B**) net ammonification rate, and (**C**) net nitrification rate). A: *A. cristatum* single species, C: *C. spinifex* single species. 3C + 1A: *C. spinifex* and *A. cristatum* in a 3:1 ratio, 2C + 2A: *C. spinifex* and *A. cristatum* in a 2:2 ratio, 1C + 3A: *C. spinifex* and *A. cristatum* in a 1:3 ratio. CK: control, LA: low-concentration ammonium nitrogen, HA: high-concentration ammonium nitrogen, LN: low-concentration nitrate nitrogen, HN: high-concentration nitrate nitrogen (*n* = 3).

The addition of a high concentration of nitrogen significantly attenuated the net mineralization rate of *C. spinifex* rhizosphere soil (*p* < 0.05) (Figure 4A). Moreover, under the 1C + 3A planting mode, the net mineralization rate of the rhizosphere soil was notably lower compared to that of the other planting modes (*p* < 0.05). In the absence of the addition of any nitrogen, different planting modes resulted in varying net mineralization rates of the rhizosphere soil: 2C + 2A > 3C + 1A > 1C + 3A. The supplementation of high concentrations of ammonium nitrogen significantly decreased the net ammonification rate in the rhizosphere soil of *C. spinifex* (*p* < 0.05) (Figure 4B). When using the 1C + 3A planting mode, the net ammonification rate in the rhizosphere soil was strikingly lower, compared to the other planting modes. In the absence of the addition of any nitrogen, the net ammonification rate of the rhizosphere soil among the different planting modes was sequenced as follows: 2C + 2A > 3C + 1A > 1C + 3A.

The net nitrification rate of the rhizosphere soil was significantly reduced under the single mode of *C. spinifex* and *A. cristatum* upon the incorporation of high concentrations of ammonium nitrogen (*p* < 0.05) (Figure 4C). In the mixed planting mode, the net nitrification rate of *C. spinifex* rhizosphere soil was significantly decreased with the supplementation of high concentrations of nitrate nitrogen.

#### 3.2.6. Abundance of Key Microbial Communities in the Soil Nitrogen Cycle in Response to the Addition of Nitrogen and Interspecies Competition

Employing qPCR assays, a quantitative assessment of the abundance levels of the *nifH*, AOA-*amoA*, AOB-*amoA*, and *nirK* genes was executed in the soil specimens derived from the five planting modes under the five distinct nitrogen levels. The amplification efficacies of the standard curves surpassed 93% (Supplementary File/Figure S10), the correlations exceeded 0.99 (Supplementary File/Figure S9), and all of the melting curves manifested as single peaks (Supplementary File/Figure S11), substantiating the validity of the curve-analysis outcomes.

**Figure 5:**
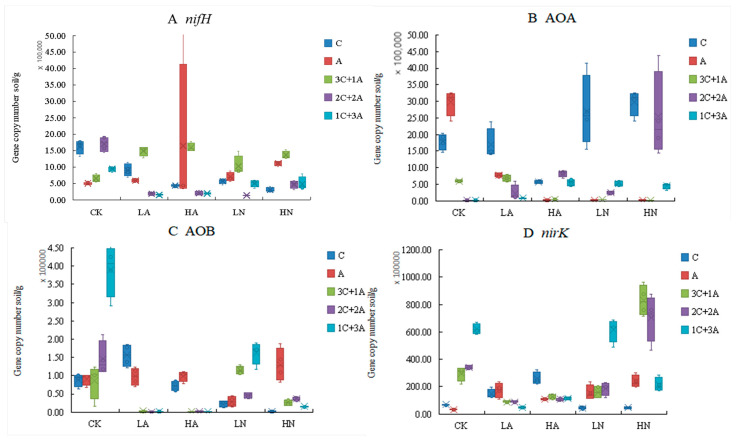
Abundance of soil nitrogen-transformation functional genes. ((**A**) nitrogen-fixing gene *nifH*, (**B**) ammonia-oxidizing archaea gene AOA, (**C**) ammonia-oxidizing bacteria AOB, and (**D**) denitrifying bacteria *nirK*). A: *A. cristatum* single species, C: *C. spinifex* single species. 3C + 1A: *C. spinifex* and *A. cristatum* in a 3:1 ratio, 2C + 2A: *C. spinifex* and *A. cristatum* in a 2:2 ratio, 1C + 3A: *C. spinifex* and *A. cristatum* in a 1:3 ratio. CK: control, LA: low-concentration ammonium nitrogen, HA: high-concentration ammonium nitrogen, LN: low-concentration nitrate nitrogen, HN: high-concentration nitrate nitrogen (*n* = 3).

Distinct variations in the *nifH* abundance were discerned among CK, LA, LN, and HN under different planting patterns with the same nitrogen-addition level (*p* < 0.05) (Figure 5A). There were significant differences in the *nifH* abundance between C, 3C + 1A, 2C + 2A, and 1C + 3A under identical planting patterns but different nitrogen-addition levels (*p* < 0.05). In a single case without any nitrogen, the abundance of *nifH* in the rhizosphere soil of *C. spinifex* was at its maximum; conversely, following nitrogen-addition treatment, the abundance of *nifH* in the rhizosphere soil was significantly reduced. In contrast, the *nifH* abundance in the rhizosphere soil of *A. cristatum* was the lowest without the addition of any nitrogen but displayed a significant increase post-treatment. In the competition modes of 2C + 2A and 1C + 3A, the abundance of *nifH* without nitrogen-addition treatment was strikingly higher than that with supplementation. On the contrary, in the competition mode of 3C + 1A, the abundance of *nifH* without nitrogen-addition treatment was significantly lower in comparison to that with treatment.

The abundance of AOA-*amoA* exhibited significant differences across various planting patterns with identical nitrogen-addition levels (*p* < 0.05) (Figure 5B). Under the single mode, and without any nitrogen-addition treatment, the abundance of AOA-*amoA* was highest in *C. spinifex* and *Agropyron cristatum*, but it significantly decreased following nitrogen treatment. Furthermore, the abundance of AOA-*amoA* under nitrate nitrogen treatment was markedly lower than that observed under ammonium nitrogen treatment. In competitive modes such as 2C + 2A and 1C + 3A, the abundance of AOA-*amoA* in the rhizosphere soil without nitrogen-addition treatment was significantly greater than that with the addition of nitrogen.

The abundance of AOB-*amoA* manifested significant differences across diverse planting patterns with uniform nitrogen-addition levels (*p* < 0.05) (Figure 5C). In the single mode, the abundance of AOB-*amoA* in *C. spinifex* was optimized under low concentrations of ammonium nitrogen, while the maximal abundance in *A. cristatum* was observed when high concentrations of nitrate nitrogen were applied. Under the competition modes of 2C + 2A and 1C + 3A, the abundance of AOB-*amoA* without nitrogen-addition treatment was significantly higher than values under the nitrogen-addition treatment. Under the 3C + 1A planting mode, the zenith abundance of AOB-*amoA* in the rhizosphere soil was observed when low concentrations of nitrate nitrogen were applied.

The abundance of *nirK* showed significant differences between different planting modes with the same nitrogen-addition level (*p* < 0.05) (Figure 5D). In the single mode, the abundance of *nirK* in *C. spinifex* was highest when low concentrations of ammonium nitrogen were applied, while the abundance of *nirK* in *A. cristatum* was highest with high concentrations of nitrate nitrogen. Under the competition modes of 3C + 1A and 2C + 2A, the highest *nirK* abundance was noted when high concentrations of nitrate nitrogen were applied.

#### 3.2.7. Network Heat Map Analysis of Key Microbial Community Abundance and Soil Environmental Factors in the Soil Nitrogen Cycle

**Figure 6:**
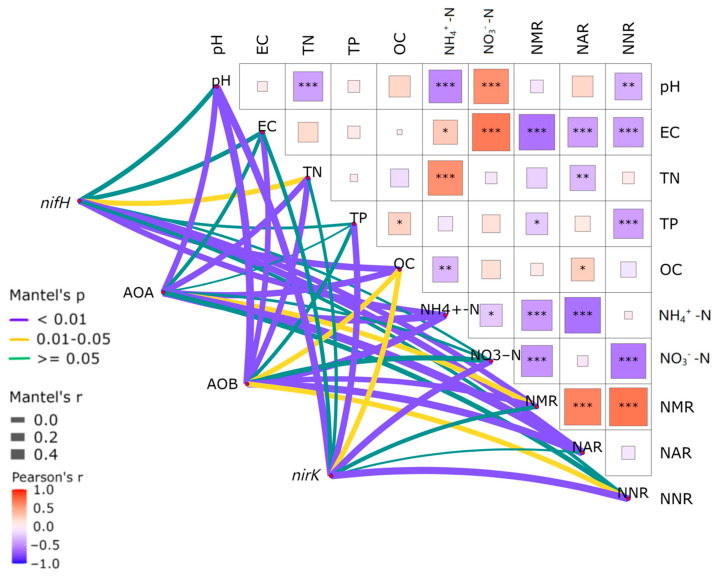
Correlation heat map analysis of rhizosphere soil environmental factors and the abundance of soil nitrogen conversion functional genes in *C. spinifex.* The right triangle diagram shows Pearson correlation coefficients associated with the physical and chemical properties of different soils and nitrogen conversion rates. See the left line diagram for Mantel test results. For Mantel analysis, we used proportional values of soil physicochemical properties and nitrogen conversion rates. Before calculating the Euclidean distance, the alpha diversity was scaled. The meanings of abbreviations for soil physical and chemical properties, functional genes, and nitrogen conversion rate are shown in the text. The line width corresponds to the partial Mantel r statistic, and the line color represents statistical significance based on 999 permutations. The environmental factors were compared in pairs. Color gradients are used to express Pearson correlation coefficients. “*” indicates a significant correlation (*p* < 0.05); “**” represents a very significant association (*p* < 0.01); “***” represents a very significant association (*p* < 0.001), and the number represents an F value. (*n* = 3).

The abundance of the *nifH* gene was significantly correlated with the organic carbon content, ammonium nitrogen content, net mineralization rate, and net ammonification rate (*p* < 0.01), as well as with the total nitrogen content (*p* < 0.01) (Figure 6). The abundance of the AOA-*amoA* gene showed a significant correlation with the pH value, total nitrogen content, organic carbon content, ammonium nitrogen content, and net ammonification rate (*p* < 0.01), as well as with the net mineralization rate (*p* < 0.05). Moreover, the abundance of the AOB-*amoA* gene was significantly correlated with the pH value, conductivity value, total nitrogen content, ammonium nitrogen content, net mineralization rate, and net ammonification rate (*p* < 0.01), as well as with the organic carbon content and net nitrification rate (*p* < 0.05). The abundance of the *nirK* gene exhibited a significant correlation with the pH value, total phosphorus content, nitrate nitrogen content, and net nitrification rate (*p* < 0.01), as well as with the organic carbon content (*p* < 0.05).According to Pearson’s correlation analysis, salient negative correlations were discerned between the pH value of the rhizosphere soil and total nitrogen content, ammonium nitrogen content, and net nitrification rate, along with a pronounced positive correlation with the nitrate nitrogen content (*p* < 0.05). There was a significant negative correlation between the conductivity value and the net mineralization rate, net ammonification rate, and net nitrification rate, and a significant positive correlation with the nitrate nitrogen content (*p* < 0.05). A strikingly significant negative correlation was observed between the total nitrogen content and ammonia nitrogen (*p* < 0.05). Additionally, a notable negative correlation was detected between the total phosphorus content and net nitrification rate (*p* < 0.05). There was a highly significant negative correlation between the ammonia nitrogen content and the net mineralization and ammonification rates (*p* < 0.05). Eventually, a significant negative correlation emerged between the nitrate nitrogen content and the net mineralization and net nitrification rates (*p* < 0.05), while a significant positive correlation was observed among the net mineralization, net ammonification, and net nitrification rates (*p* < 0.05).

#### 3.2.8. Structural Equation Model (SEM)

**Figure 7:**
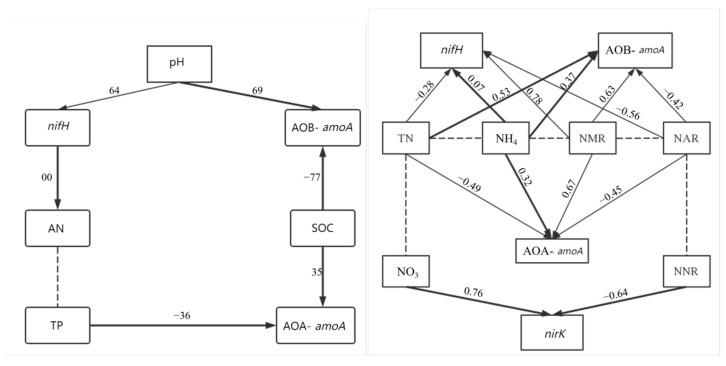
Structural equation modeling (SEM) describing how the invasion of *C. spinifex* affected the key microbial communities involved in nitrogen conversion by altering the soil environment, and how nitrogen conversion rates were influenced through both nitrogen fixation and non-nitrogen-fixation processes. The size of the effect was standardized, and the number next to the arrow represents the standardized regression coefficient. The black solid line with arrow and the dashed line represent the positive path coefficient and the insignificant path coefficient, respectively. Since the implicit covariance matrix of the model was not significantly different from the original covariance matrix (v2 test, *p* > 0.05), the model cannot be rejected.

The X^2^ test indicated that there was no significant difference between the implied covariance matrix of the model and the original covariance matrix, thereby affirming the reliability of our SEM (*p* > 0.05) (Figure 7). The SEM of the field experiments showed that the invasion of *C. spinifex* affects the abundance of *nifH* and AOB-*amoA* by regulating the soil pH of the rhizosphere soil. In particular, this invasion affects the abundance of *nifH* by exerting an influence on the AN content, while it influences the abundance of AOB-*amoA* by causing alterations in the SOC content. Moreover, the invasion indirectly affects the abundance of AOA-*amoA* by affecting the TP and SOC contents.

The outcomes of the SEM derived from the pot experiment demonstrated that *C. spinifex* exerts an influence on the gene abundance of *nifH*, AOA-*amoA,* and AOB-*amoA* by affecting the TN content, NH_4_^+^ content, NMR, and NAR. Additionally, *C. spinifex* impacts the gene abundance of *nirK* by virtue of its effect on the NO_3_^−^ content and NNR.

## 4. Discussion

### 4.1. Cenchrus Spinifex Exhibits a Competitive Growth Strategy Compared to Native Plants

Interspecific competition between invasive and native plant species is predominantly and clearly reflected in the biomass allocation of plants [35]. When soil resources are abundant, invasive plants demonstrate a competitive advantage over native plants. In this study, invasion consistently engendered a reduction in the plant height of the native species, in accordance with Florianová et al.’s findings, which highlighted that *Impatiens parviflora* invasion occasionally decreased plant height among native plants in the invaded area [36]. We observed a significant decrease in the biomass of *Agropyron cristatum*, a native plant, subsequent to the invasion of *C. spinifex*. Previous studies have demonstrated that the existence of the invasive plant *Flaveria bidentis* (L.) leads to reduced stomatal conductance and transpiration rate in native plants on account of the shading effects. Additionally, this inhibits the plants’ ability to acquire light resources, ultimately culminating in attenuated growth indicators for native plants [37]. We postulate that under circumstances of resource opulence, plants disproportionately allocate additional resources to their supra-terrestrial structures. The increased coverage of *C. spinifex* could enhance its competitive superiority and impede native plant *A. cristatum’s* access to light resources, creating a shading effect that inhibits the growth of *A. cristatum* and significantly reduces its biomass. Prior studies have demonstrated the beneficial effects of even marginal increases in nitrogen content on non-native species [38]. In this study, the supplementation of low concentrations of nitrate facilitated biomass accumulation in *C. spinifex*, while the addition of high concentrations of ammonium inhibited its growth (plant height and biomass data were presented). This phenomenon may be attributed to the potential facilitation of growth, survival, and reproduction in *C. spinifex* by nitrogen deposition; however, it also resulted in increased soil osmotic pressure, leading to physiological drought and adversely affecting water absorption. Consequently, an elevated concentration of added nitrogen restrained the growth of *C. spinifex*.

### 4.2. C. spinifex Prefers Nitrogen Absorption for Successful Invasion

The discrepancy in competitiveness between *C. spinifex* and native *A. cristatum* is predominantly governed by soil nitrogen, as indicated by our data. We propose that modifications in the soil nitrogen environment milieu constitute one of the mechanisms through which invasive plants exert their influence on invaded ecosystems [39]. Fluctuations in nutrient content within the soil can likely be attributed to disparities in plant uptake and utilization of nitrogen elements [40]. Our findings demonstrated a considerable increase in the total nitrogen content in rhizosphere soil due to elevated levels of ammonia; however, the incursion of *C. spinifex* was linked to a notable reduction in both the total nitrogen and the ammonium contents, concomitant with an escalation in nitrate levels. Previous studies have confirmed that invasive species exhibit preferences for specific nutrient types; for instance, *Spartina alterniflora* invasions prioritize the uptake of ammonium over nitrate, which is consistent with our findings [41]. Compared to other species, the nitrogen content in the leaves and fine roots of *Robinia pseudoacacia* demonstrate a more pronounced correlation with the plant’s nitrogen-fixing capability [42]. The invasion of *Spartina alterniflora* gives rise to an increase in the soil nitrate content and organic matter accumulation [43]. A meta-analysis further demonstrated that invasive species possess a higher nutrient absorption efficiency than native species within nutrient-deficient milieus [1]. Concerning nitrogen-acquisition strategies, both invasive and native species present homologous patterns in their assimilation of inorganic nitrogen. The mechanisms underpinning the augmented nitrogen uptake by invasive species are multifaceted, encompassing root morphology, greater belowground biomass allocation, and enhanced flexibility in transitioning between diverse nitrogen sources [44]. Comparing NH_4_^+^ or NO_3_^−^ as a sole nitrogen source, *C. spinifex* preferred NH_4_^+^. This observation suggests that *C. spinifex* selectively absorbs and utilizes NO_3_^−^ in the soil, thereby mitigating the accumulation of available nitrogen. We hypothesize that this preference might act as a strategic mechanism for *C. spinifex,* facilitating the efficient acquisition of inorganic nitrogen nutrients. Previous studies have demonstrated that assimilating NH_4_^+^ into essential amino acids requires less energy compared to NO_3_^−^ [45].

### 4.3. Cenchrus spinifex Changes the Soil Environment and Affects the Key Microorganisms of Nitrogen Transformation, Thus Impacting the Nitrogen Cycle

The field experiments highlighted the fact that the invasion of *C. spinifex* has substantial ramifications on the soil environment, engendering an increase in the soil total nitrogen content and a decrease in the soil phosphorus content. Moreover, this invasive species exerts substantial effects on the soil microbial community, which plays a crucial role in nitrogen cycling. Specifically, invasion reduces the gene abundance of *nifH*, AOA-*amoA*, and *nirK* while increasing the gene abundance of AOB-*amoA*. Additionally, alterations in the soil pH, alkali-hydrolyzed nitrogen, organic carbon, and total phosphorus contents are key factors influencing the microbial community involved in nitrogen cycling. The total nitrogen content in the soil with a high concentration of ammonium nitrate was significantly higher in the planting patterns of C, 3C + 1A, and 2C + 2A than in the planting pattern of 1C + 3A. Consequently, by modifying both the physical and chemical properties of the soil, as well as its microbial composition, invasive plants could influence the rates of nitrogen transformation and subsequently impact interspecific competition and responses to nitrogen-addition treatments. Notably, our study revealed that high concentrations of added nitrogen significantly decreased the net mineralization rates within *C. spinifex*-rhizosphere soils. The presence of the invasive plant *Ambrosia artemisiifolia* significantly reduces the net mineralization rate of soil under elevated concentrations of ammonium and nitrate [46]. Similarly, *Eupatorium adenophorum* leads to a significant reduction in the soil-net-mineralization rate, particularly under high levels of ammonium and nitrate, corroborating the findings of this study [47]. These outcomes further validate our initial hypothesis that elevated concentrations of ammonium or nitrate might exert detrimental effects on soil microbial communities and ecosystems, consequently diminishing the net rhizosphere soil-mineralization rate associated with invasive plants. We observed that the rhizosphere soil-net-mineralization rate reached its nadir when the competition pattern between *C. spinifex* and *A. cristatum* was 1C + 3A. Furthermore, previous investigations have demonstrated that in grasslands, the lowest soil-net-mineralization rate transpires when the relative competition intensity between invasive and native plants is at a ratio of 1:3 [48]. It is worth noting that the addition of high concentrations of ammonium in this study significantly curtailed the net ammonification rate of the rhizosphere soil. Specifically, the infusion of 200 mg·L^−1^ of ammonium engendered a pronounced decrease in the net ammonification rate of *Microstegium vimineum* rhizosphere soil [49], which was in accordance with our findings. The repression of soil microbial activity resulting from high concentrations of ammonium could be construed as a contributory element. Additionally, the competition between invasive and native plants also influenced the net ammoniation rate in the soil. Invasive species were endowed with the capability to sequester ammonium from the soil, leading to a reduction in available nitrogen content and subsequently impinging upon the growth and metabolic characteristics of soil microorganisms. Our findings imply that interspecific rivalry attenuates the net nitrification rate of rhizosphere soil under high concentrations of nitrate. Previous studies have attested that elevated levels of nitrate in forest ecosystems could aggravate the competition between coniferous and broadleaved forests, resulting in a decrease in the net nitrification rate in rhizosphere soil [50].

Our SEM analysis demonstrated the influence *C. spinifex* exerted on AOA-*amoA* and AOB-*amoA* expression through the plant’s nitrogen-absorption preference. In contrast, *Spartina alterniflora* has a proclivity for NO_3_^−^-N over NH_4_^+^-N [51]. These discoveries further insinuate that the growth of *C. spinifex* primarily relies on soil nitrogen availability, suggesting that nitrogen-rich soils might be more prone to invasion by this species, compared to nitrogen-deficient soils, which aligns with prevalent observations concerning invasive plants [52].

The pivotal microorganisms engaged in soil nitrogen transformation serve as constraining factors for the process. Our investigation highlighted a substantial attenuation in the abundance of the *nifH* gene in the rhizosphere soil of *C. spinifex* under the single, 2C + 2A, and 1C + 3A cropping patterns upon nitrogen supplementation, consistent with the field observations. It has been noted that adding nitrogen leads to a pronounced reduction in the *nifH* gene abundance within the rhizosphere soil of desert grassland [53]. We postulate that adding nitrogen could suppress the growth and metabolic activity of nitrogen-fixing microorganisms in soil by modifying both the structure and metabolic characteristics of the soil microbial community, leading to a reduction in *nifH* gene abundance. Interestingly, contrary to field observations, we discerned a decline in the abundance of AOA-*amoA* and AOB-*amoA* following the addition of nitrogen. Furthermore, regardless of whether native species coexist with *Mikania micrantha* or not, the rhizosphere soil associated with invasive *Mikania micrantha* exhibits higher concentrations of NO_3_^−^-N compared to the rhizosphere soil of the invasion site, signifying distinct dissimilarities in soil microbial communities intimately associated with nitrogen transformation [22]. Nitrification constitutes a crucial process within the global nitrogen cycle and is inextricably interwoven with nitrogen mineralization and biological fixation and loss. The existence and profusion of ammonia-oxidizing microorganisms constitute limiting factors governing soil nitrification [54]. It is widely acknowledged that plant invasion increases the soil NO_3_^−^-N content, which is attributed to substantial alterations in the composition and structure of ammonia-oxidizing microbial communities [55]. We hypothesize that the administration of nitrogen treatment might instigate alterations in soil pH, thereby influencing the growth and metabolic activity of ammonia-oxidizing archaea [56]. Moreover, an excessive supply of ammonia could potentially repress AOB-*amoA* expression [57]. The abundance of *nirK* in this study tended to increase following the addition of nitrogen, which can potentially be attributed to the enhanced organic matter content in soil induced by nitrogen-addition treatment. This subsequently enhanced the availability of carbon and energy sources for bacteria, thereby facilitating the expression and abundance of the *nirK* gene [58]. To substantiate our initial hypothesis, our structural equation model confirmed that TN, NH_4_^+^, NMR, and NAR exerted affirmative effects on the key nitrogen-transforming microorganisms in the rhizosphere soil of *C. spinifex*. Among these factors, *nifH*, AOA, and AOB played a pivotal role in this process, conforming to previous research findings. Moreover, we observed significant correlations between the gene abundance of key nitrogen-cycling microorganisms and environmental factors in the rhizosphere soil during plant invasion. Specifically, soil pH exhibits a significant positive correlation with the ammonium content while demonstrating a markedly negative correlation with the nitrate content [59]. In this study, a significant negative correlation was detected between the rhizosphere soil conductivity and the rates of soil net mineralization, ammonification, and nitrification. The escalation in rhizosphere soil conductivity hampers the microbial activity associated with nitrogen transformation in the soil, leading to a reduction in the rates of net mineralization, ammonification, and nitrification [60]. Additionally, a highly significant negative correlation was identified among the rates of net mineralization, ammonification, and nitrification in rhizosphere soil. Competitive interactions among different nitrogen-transformation processes are apparent in rhizosphere soil, with particular emphasis on the influence of net mineralization rate on both net ammonification rate and net nitrification rate [61].

The results further suggest that *C. spinifex* could influence soil nitrogen transformation by altering the composition of microorganisms actively involved in soil nitrogen cycling, enhancing its competitive advantages in terms of nitrogen uptake, transformation rate, and growth, as well as accumulating soil nitrogen in a preferred form for absorption and utilization. Consequently, these adaptations improve its ability to outcompete native plants for resources and have facilitated the successful invasion of *C. spinifex* as a dominant population in the sandy grassland ecosystem in Horqin. This study highlights the importance of nitrogen cycling in plant competition and invasion, providing clues for us to understand the response characteristics and feedback mechanisms of rhizosphere soil microbial communities to diverse nitrogen-addition forms during the competition for nitrogen resources between invasive plants and native symbiotic plants.

## 5. Conclusions

Our findings highlight the significance of nitrogen cycling in plant competition and invasion. The results demonstrated that the invasion of *Cenchrus spinifex* could bring about substantial modifications in soil conditions, while different forms of nitrogen application and interspecific competition also exert substantial impacts on soil environmental factors. Under the influence of *C. spinifex* invasion, soil pH, ammonium nitrate, total phosphorus, and organic carbon emerged as predominant determinants for alterations in the key microbial communities involved in nitrogen cycling. Moreover, *C. spinifex* invasion reconfigured plant growth attributes and nutrient profiles while exhibiting varying effects on diverse key microorganisms participating in soil nitrogen cycling. The addition of nitrogen and interspecific competition significantly impacted plant growth characteristics. The invasion of *C. spinifex* impeded the growth of the native plant *Agropyron cristatum*, with a more pronounced effect observed under a low nitrate concentration. Furthermore, nitrogen supplementation and interspecific competition yielded distinct effects on the key microorganisms involved in soil nitrogen cycling. In response to the addition of nitrogen and interspecific competition, soil pH, total nitrogen, organic carbon, and ammonium content emerged as the primary drivers of changes in soil microbial communities associated with nitrogen transformation. Furthermore, a low concentration of added ammonium significantly enhanced the abundance of functional genes related to soil nitrogen transformation.

## Figures and Tables

**Table 1:** Impact of *C. spinifex* invasion on physicochemical properties of the soil.

	LD	BY	HD	SY
TN (g∙kg^−1^)	0.20 ± 0.01 ^ab^	0.18 ± 0.01 ^b^	0.21 ± 0.01 ^a^	0.20 ± 0.01 ^ab^
NH_4_^+^-N (mg∙kg^−1^)	1.80 ± 1.05	0.69 ± 0.04	2.17 ± 0.39	1.11 ± 0.30
AN (mg∙kg^−1^)	20.67 ± 2.05 ^ab^	21.33 ± 0.33 ^a^	16.92 ± 0.65 ^b^	23.92 ± 1.17 ^a^
NO_3_^+^-N (mg∙kg^−1^)	1.75 ± 0.74	1.51 ± 0.16	3.06 ± 0.53	3.02 ± 0.30
TP (g∙kg^−1^)	0.42 ± 0.01 ^a^	0.32 ± 0.05 ^ab^	0.32 ± 0.04 ^ab^	0.27 ± 0.02 ^b^
AP (mg·kg^−1^)	0.39 ± 0.02 ^ab^	0.47 ± 0.03 ^a^	0.43 ± 0.04 ^ab^	0.32 ± 0.05 ^b^
SOC (g∙kg^−1^)	1.50 ± 0.06 ^a^	1.03 ± 0.06 ^bc^	0.99 ± 0.11 ^c^	1.36 ± 0.15 ^ab^
EC (S∙m^−1^)	13.34 ± 1.63 ^ab^	15.21 ± 1.11 ^a^	10.73 ± 0.95 ^b^	10.36 ± 0.42 ^b^
pH	5.76 ± 0.18	5.65 ± 0.11	5.56 ± 0.04	5.59 ± 0.15
NNR (mg·g^−1^·d^−1^)	0.40 ± 0.12 ^b^	0.96 ± 0.16 ^a^	0.92 ± 0.12 ^a^	0.79 ± 0.14 ^ab^
NMR (mg·g^−1^·d^−1^)	1.22 ± 0.06	1.04 ± 0.14	1.23 ± 0.06	1.58 ± 0.35
NAR (mg·g^−1^·d^−1^)	0.83 ± 0.06 ^a^	0.08 ± 0.24 ^b^	0.31 ± 0.18 ^ab^	0.79 ± 0.29 ^ab^

LD refers to bare land, BY refers to the sample land of the local plant community, HD refers to the sample land of mixed communities of *C. spinifex* and local plants, and SY refers to the invasion sample land composed of patches inlaid by monodominant communities of *C. spinifex*. TN: total nitrogen, AN: alkali-hydrolyzable nitrogen, NH_4_^+^-N: ammonium nitrogen, NO_3_^+^-N: nitrate nitrogen, TP: total phosphorus, AP: available phosphorus, SOC: soil organic carbon, EC: soil electrical conductivity, NMR: net mineralization rate, NAR: net ammonification rate, NNR: net nitrification rate. The figures in the table represent the average value ± standard error. Different lowercase letters indicate significant differences between different invasive habitats during the same growth period (*p* < 0.05). Different capital letters indicate significant differences between different growth periods in the same invasive habitat (*p* < 0.05).

## Data Availability

The raw data supporting the conclusions of this article will be made available by the authors on request.

## Supplementary Materials

The following supporting information can be downloaded at: https://www.mdpi.com/article/10.3390/microorganisms12112120/s1. Figure S1: Schematic representations of four distinct levels of habitat invasion; Figure S2: Design drawing of pot experiment; Figure S3: Standard curve of nitrogen cycle functional genes by real-time flourescence quantitative PCR; Figure S4: Amplification curve of nitrogen cycle functional genes by real-time flourescence quantitative PCR; Figure S5: Melting curve of nitrogen cycle functional genes by real-time flourescence quantitative PCR; Figure S6: Plant height (A: Plant height of *A. cristatum* B: plant height of *C. spinifex*); Figure S7: Plant biomass (A: total biomass of *A. cristatum* B: total biomass of *C. spinifex*); Figure S8: Physical and chemical properties of rhizosphere soil (A: TN, B: TP, C: soil pH, D: EC, E: SOC, F: NH_4_^+^-N, G: NO_3_^+^-N); Figure S9: Standard curve of nitrogen cycle functional genes by real-time flourescence quantitative PCR; Figure S10: Amplification curve of nitrogen cycle functional genes by real-time flourescence quantitative PCR; Figure S11: Melting curve of nitrogen cycle functional genes by real-time flourescence quantitative PCR; Table S1: Basic physical and chemical properties of soil in the test site; Table S2: Primers used for PCR amplification; Table S3: Effects and interactions of plant species, planting patterns and nitrogen addition on plant height (cm·plant^−1^) and biomass (g·plant^−1^).

## References

[1] Sardans J., Bartrons M., Margalef O., Gargallo-Garriga A., Janssens I.A., Ciais P., Obersteiner M., Sigurdsson B.D., Chen H.Y.H., Peñuelas J. (2017). Plant invasion is associated with higher plant–soil nutrient concentrations in nutrient-poor environments. Glob. Chang. Biol..

[2] Bradley B.A., Blumenthal D.M., Wilcove D.S., Ziska L.H. (2010). Predicting plant invasions in an era of global change. Trends Ecol. Evol..

[3] Wang J.J., He L.L. (2022). Geographical distribution and main environmental impact factors of *Cenchrus spinifex* in Liaoning Province. Mod. Agric..

[4] Sun Z.L., Shu Q., Gao K., Zhou L.Y., Tian X., Guo F.C. (2020). Invasion Status, Adaptive Mechanism and Control Strategy of Field Sandbur: A Review. Acta Agrestia Sin..

[5] Ma J.B., Zhang Y.L., Tian X., Zhou L.Y. (2020). Studies on physiological adaptability of *Cenchrus pauciflorus* in different growth periods of Horqin sandy land. Grassl. Turf..

[6] Mark M.C., D’Antonio C.M. (2003). Exotic grasses alter controls over soil nitrogen dynamics in a Hawaiian woodland. Ecol. Appl..

[7] Fei S., Phillips J., Shouse M. (2014). Biogeomorphic impacts of invasive species. Annu. Rev. Ecol. Evol. Syst..

[8] Hulme P.E., Pysek P., Jarosík V., Pergl J., Schaffner U., Vilà M. (2013). Bias and error in understanding plant invasion impacts. Trends Ecol. Evol..

[9] Liao M., Xie X.M., Peng Y., Chai J.J., Chen N. (2013). Characteristics of soil microbial community functional and structure diversity with coverage of *Solidago Canadensis* L.. J. Cent. South Univ..

[10] Kaur R., Malhotra S., Inderjit. (2012). Effects of invasion of mikania micrantha on germination of rice seedlings, plant richness, chemical properties and respiration of soil. Biol. Fertil. Soils.

[11] Sharma G.P., Raghubanshi A.S. (2009). Lantana invasion alters soil nitrogen pools and processes in the tropical dry deciduous forest of India. Appl. Soil Ecol..

[12] Huang J., Xu X., Wang M., Nie M., Qiu S., Wang Q., Quan Z., Xiao M., Li B. (2016). Responses of soil nitrogen fixation to Spartina alterniflora invasion and nitrogen addition in a Chinese salt marsh. Sci. Rep..

[13] Lu J.Z., Qiu W., Chen J.K., Li B. (2005). Impact of invasive species on soil properties: Canadian goldenrod (*Solidago canadensis*) as a case study. Chin. Biodivers..

[14] Yang G., Liu N., Lu W., Wang S., Kan H.M., Zhang Y.J., Xu L., Chen Y.L. (2014). The interaction between arbuscular mycorrhizal fungi and soil phosphorus availability influences plant community productivity and ecosystem stability. J. Ecol..

[15] Geldner N., Salt D.E. (2014). Focus on roots. Plant Physiol..

[16] Hou Q.C., Fen Y.L., Zhou Y.J., Ao Y.M., Chen C.X., Xin Y.J., Wang Q.G., Yan G.Y. (2022). Major hypotheses on plant invasion mechanisms. Chin. J. Appl. Ecol..

[17] Chang E.H., Chiu C.Y. (2015). Changes in soil microbial community structure and activity in a cedar plantation invaded by moso bamboo. Appl. Soil Ecol..

[18] Yan J.F., Wang L., Tsang Y.F., Qian L.W., Fu X.H., Sun Y., Wu P.F. (2020). Conversion of organic carbon from decayed native and invasive plant litter in jiuduansha wetland and its implications for soc formation and sequestration. J. Soil Sediments.

[19] Klironomos J.N. (2002). Feedback with soil biota contributes to plant rarity and invasiveness in communities. Nature.

[20] Lin G., He Y., Lu J., Chen H., Feng J. (2021). Seasonal variations in soil physicochemical properties and microbial community structure influenced by spartina alterniflora invasion and kandelia obovata restoration. Sci. Total Environ..

[21] Li Q., Wan F., Zhao M. (2022). Distinct soil microbial communities under Ageratina adenophora invasions. Plant Biol..

[22] Kuypers M.M.M., Marchant H.K., Kartal B. (2018). The microbial nitrogen-cycling network. Nat. Rev. Microbiol..

[23] Rodrigues R.R., Pineda R.P., Barney J.N., Nilsen E.T., Barrett J.E., Williams M.A. (2015). Plant invasions associated with change in root-zone microbial community structure and diversity. PLoS ONE.

[24] Kazakou E., Dimitrakopoulos P.G., Baker A.J., Reeves R.D., Troumbis A.Y. (2008). Hypotheses, mechanisms and trade-offs of tolerance and adaptation to serpentine soils: From species to ecosystem level. Biol. Rev..

[25] Piper C.L., Lamb E.G., Siciliano S.D. (2015). Smooth brome changes gross soil nitrogen cycling processes during invasion of a rough fescue grassland. Plant Ecol..

[26] Dassonville N., Guillaumaud N., Piola F., Meerts P., Poly F. (2011). Niche construction by the invasive asian knotweeds (species complex Fallopia): Impact on activity abundance and community structure of denitrifiers and nitrifiers. Biol. Invasions.

[27] Chen B.M., Wei H.J., Chen W.B., Zhu Z.C., Yuan Y., Zhang Y.L., Lang Z.G. (2018). Effects of plant invasion on soil nitrogen transformation processes and its associated microbes. Chin. J. Plant Ecol..

[28] Bao S.D. (2000). Soil agronomy analysis. Soil Chemical Analysis for Agriculture.

[29] Armas C., Pugnaire F.I. (2009). Ontogenetic shifts in interactions of two dominant shrub species in a semi-arid coastal sand dune system. J. Veg. Sci..

[30] Chiuffo M.C., MacDougall A.S., Hierro J.L. (2015). Native and non-native ruderals experience similar plant-soil feedbacks and neighbor effects in a system where they coexist. Oecologia.

[31] Schöb C., Armas C., Pugnaire F.I. (2013). Direct and indirect interactions co-determine species composition in nurse plant systems. Oikos.

[32] Ke X.B., Angel R., Lu Y.H., Conrad R. (2013). Niche Differentiation of ammonia oxidizers and nitrite oxidizers in rice paddy soil. Environ. Microbiol..

[33] Bremer E., Kuikman P. (1997). Influence of competition for nitrogen in soil on net mineralization of nitrogen. Plant Soil.

[34] Bentler P.M., Bonett D.G. (1980). Significance tests and goodness of fit in the analysis of covariance structures. Psychol. Bull..

[35] Yan B.J., Ji Z.H., Fan B., Wang X.M., He G.X., Shi L.T., Liu G.C. (2016). Plants adapted to nutrient limitation allocate less biomass into stems in an arid-hot grassland. New Phytol..

[36] Florianová A., Münzbergová Z. (2017). Invasive impatiens parviflora has negative impact on native vegetation in oak-hornbeam forests. Flora.

[37] Qi X.X., Zhang S.Y., Lin F., Zhang L.L., Yang D.L., Wang H. (2019). Effects of Flaveria bidentis on plant communities and soil microbial communities in different invasions. Acta Ecol. Sin..

[38] Ren G.Q., Yang H.Y., Li J., Prabakaran K., Dai Z.C., Wang X.P., Jiang K., Zou C.B., Du D.L. (2022). Additive effects of warming and nitrogen addition on the performance and competitiveness of invasive *Solidago canadensis* L.. Plant Species Biol..

[39] Jin B., Yan H., Zhang W., Zeng C. (2017). Contents and Storages of Various Forms of Nitrogen in Soils of Wetlands in the Min River Estuary under Spartina alterniflora Invasion. Wetl. Sci..

[40] Stefanowicz A.M., Stanek M., Nobis M., Zubek S. (2017). Few effects of invasive plants Reynoutria japonica, Rudbeckia laciniata and Solidago gigantea on soil physical and chemical properties. Sci. Total Environ..

[41] Qing H., Cai Y., Xiao Y., Yao Y.H., An S. (2015). Nitrogen Uptake and Use Efficiency of Invasive Spartina alterniflora and Native Phragmites australis: Effect of Nitrogen Supply. Clean Soil Air Water J. Sustain. Environ. Saf..

[42] Salpagarova F.S., Van L.S.P., Onipchenko V.G. (2014). Nitrogen content in fine roots and the structural and functional adaptations of alpine plants. Biol. Bull. Rev..

[43] Unkovich M., Pate J.S., Unkovich M., Pate J., Mcneill A. (2001). Assessing N_2_ fixation in annual legumes using ^15^N natural abundance. Stable Isotope Techniques in the Study of Biological Processes and Functioning of Ecosystems.

[44] Hewins D.B., Hyatt L.A. (2010). Flexible N uptake and assimilation mechanisms may assist biological invasion by Alliaria petiolata. Biol. Invasions.

[45] Luckhart S., Riehle M.A. (2009). Introduction to marine biogeochemistry. Dev. Comp. Immunol..

[46] Sun Y., Roderick G.K. (2019). Rapid evolution of invasive traits facilitates the invasion of common ragweed, ambrosia artemisiifolia. J. Ecol..

[47] Liao M., Xie X.M., Peng Y., Ma A.L. (2011). Changes of Soil Microbiological Characteristics After *Solidago canadensis* L.. Invasion. Agric. Sci. China.

[48] Wang J.W., Wang J.L., Wang J., Li W.H., Zhang C.B. (2011). Effects of *Solidago canadensis* invasion on soil enzyme activities. Plant Nutr. Fertil. Sci..

[49] Teng Q.M., Lu X.N., Zhang Q.Q., Cai L.L., Sardar M.F., Li Y.F., Abbas T., Li Y., Chang S.X., Li Y.C. (2023). Litterfall quality modulates soil ammonium and nitrate supply through altering microbial function in bamboo encroachment of broadleaf forests. Geoderma.

[50] Shen J.P., Zhang L.M., Zhu Y.G., Zhang J.B., He J.Z. (2008). Abundance and composition of ammonia, xidizing bacteria and ammonia, xidizing archaea communities of an alkaline sandy loam. Environ. Microbiol..

[51] Chen W.B., Chen B.M. (2019). Considering the preferences for nitrogen forms by invasive plants: A case study from a hydroponic culture experiment. Weed Res..

[52] Morais M.C., Oliveira P., Marchante H., Freitas H., Marchante E. (2019). Is richer always better? Consequences of invaded N-rich soils for the early growth of a native and an invasive species. Flora.

[53] Yang K., Luo S., Hu L., Chen B., Xie Z., Ma B., Ma W., Du G., Ma X., Le Roux X. (2020). Responses of soil ammonia-oxidizing bacteria and archaea diversity to n, p and np fertilization: Relationships with soil environmental variables and plant community diversity. Soil Biol. Biochem..

[54] Kowalchuk G.A., Stephen J.R. (2001). Ammonia-Oxidizing Bacteria: A Model for Molecular Microbial Ecology. Annu. Rev. Microbiol..

[55] Shannon-Firestone S., Reynolds H.L., Phillips R.P. (2015). The role of ammonium oxidizing communities in mediating effects of an invasive plant on soil nitrification. Soil Biol. Biochem..

[56] Bei S., Zhang Y.L., Li T.T., Christie P., Li X.L., Zhang J.L. (2018). Response of the soil microbial community to different fertilizer inputs in a wheat-maize rotation on a calcareous soil. Agric. Ecosyst. Environ..

[57] Di H.J., Cameron K.C. (2002). Nitrate leaching in temperate agroecosystems: Sources factors and mitigating strategies. Nutr. Cycl. Agroecosyst..

[58] Bru D., Sarr A., Philippot L. (2007). Relative abundances of proteobacterial membrane-bound and periplasmic nitrate reductases in selected environments. Appl. Environ. Microbiol..

[59] Carey C.J., Dove N.C., Beman J.M., Hart S.C., Aronson E.L. (2016). Meta-analysis reveals ammonia-oxidizing bacteria respond more strongly to nitrogen addition than ammonia-oxidizing archaea. Soil Biol. Biochem..

[60] Li W., Li Y., Lv J., He X., Wang J., Teng D., Jiang L., Wang H., Lv G. (2021). Rhizosphere effect alters the soil microbiome composition and C, N transformation in an arid ecosystem. Appl. Soil Ecol..

[61] Wang J., Shi X., Zheng C., Suter H., Huang Z. (2020). Different responses of soil bacterial and fungal communities to nitrogen deposition in a subtropical forest. Sci. Total Environ..

