# Supplementary material for: Cenchrus spinifex Invasion Alters Soil Nitrogen Dynamics and Competition
Source: Microorganisms. 2024 Oct 23;12(11):2120. doi: 10.3390/microorganisms12112120 (PMC11596749; doi:10.3390/microorganisms12112120)
Supplement: Supplementary file 1 [file microorganisms-12-02120-s001.zip › microorganisms-3207609-supplementary.pdf]

Cenchrus spinifex Invasion Alters Soil Nitrogen Dynamics and Competition

Meng Meng [1,†], Baihui Ren [1,*,†], Jianxin Yu [1], Daiyan Li [1], Haoyan Li [1], Jiahuan Li [1], Jiyun Yang [1], Long Bai [1] and Yulong Feng [2,*]

[1] College of Horticulture, Shenyang Agricultural University, Shenyang 110866, China; 2022220409@stu.syau.edu.cn (M.M.)

[2] Liaoning Key Laboratory for Biological Invasions and Global Changes, College of Bioscience and Biotechnology, Shenyang Agricultural University, Shenyang 110866, China

* Correspondence: bhren@syau.edu.cn (B.R.); fyl@syau.edu.cn (Y.F.)

† These authors have contributed equally to this work.

The following Supporting Information is available for this article:

Table S1 Basic physical and chemical properties of soil in the test site

pH	Soilorganic carbon ($g\cdot kg^{-1}$)	Total nitrogen ($g\cdot kg^{-1}$)	Total phosohorus ($g\cdot kg^{-1}$)	NH_4^+-N ($mg\cdot kg^{-1}$)	NO_3^--N ($mg\cdot kg^{-1}$)	Electrical conductivity ($S\cdot m^{-1}$)
5.74	1.25	0.12	0.31	3.17	5.32	44.85

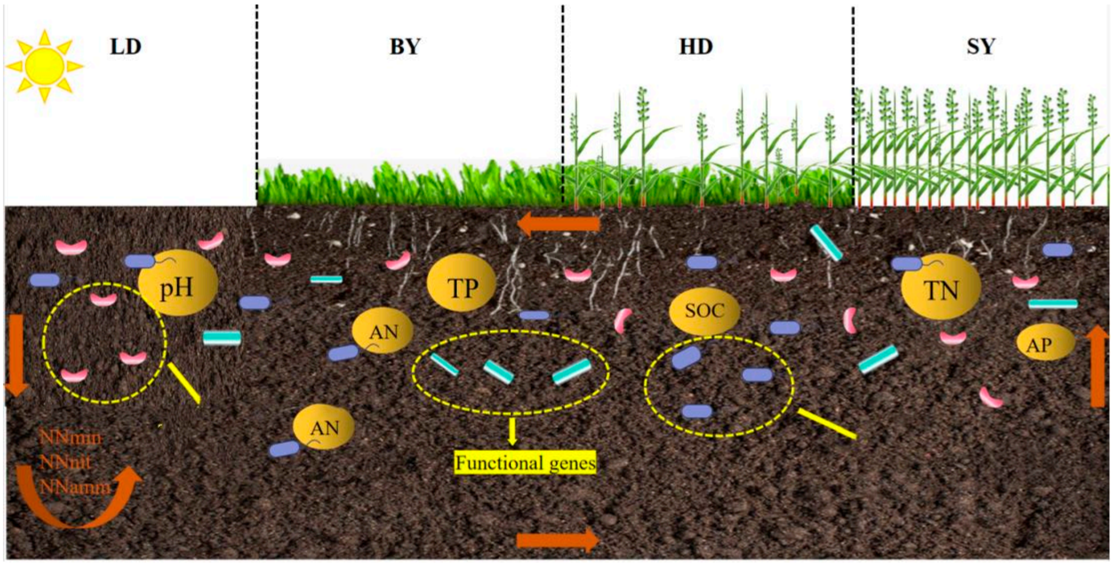

Figure S1 Schematic representations of four distinct levels of habitat invasion

Table S2 Primers used for PCR amplification

Name	Types	Sequence (5'-3')	PCR Products(bp)
nifH	Forward Primer	AAAGGYGGWATCGGYAARTCCACCAC	432
	Reverse Primer	TTGTTSGCSGCRTACATSGCCATCAT	
AOA-*amoA*	Forward Primer	GGGGTTTCTACTGGTGGT	491
	Reverse Primer	CCCCTCKGSAAAGCCTTCTTC	
AOB-*amoA*	Forward Primer	STAATGGTCTGGCTTAGACG	635
	Reverse Primer	CACCGTTTACTGCCAGGACT	
nirK	Forward Primer	GGMATGGTKCCSTGGCA	514
	Reverse Primer	GCCTCGATCAGRTTRTGG	

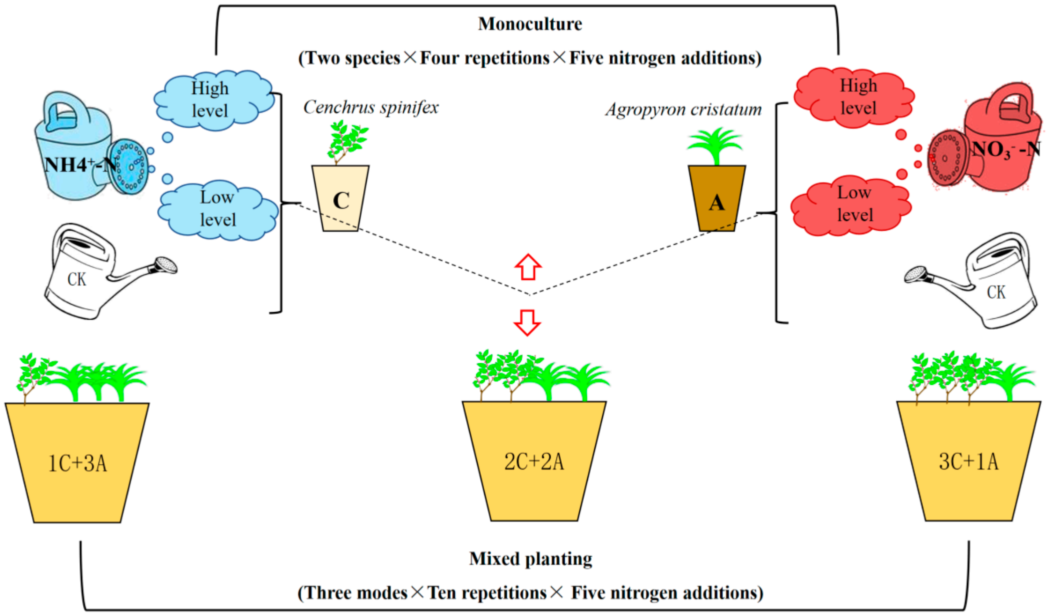

Figure S2 Design drawing of pot experiment

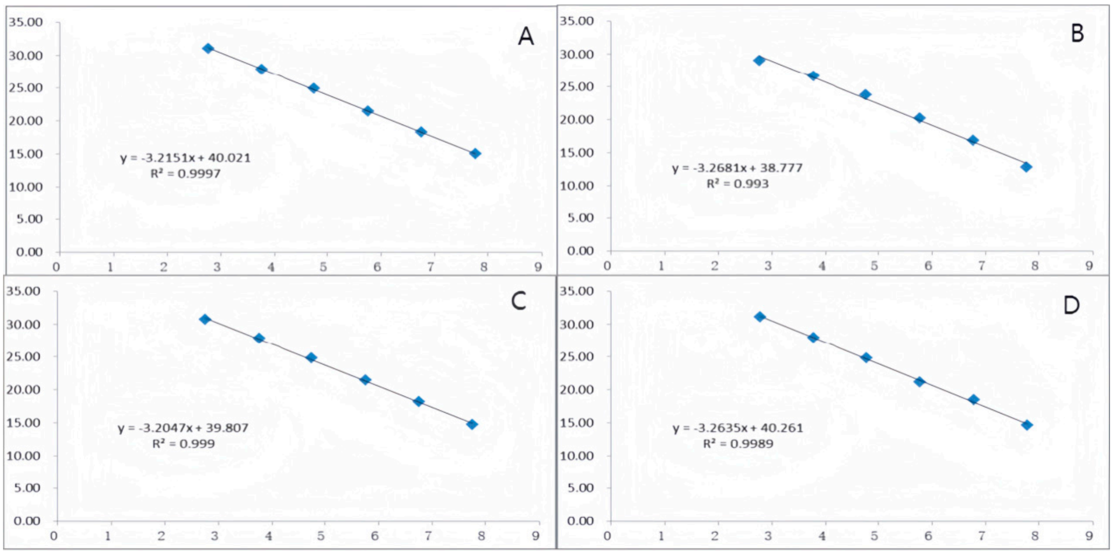

Figure S3 Standard curve of nitrogen cycle functional genes by real-time flourescence quantitative PCR

Footnote: A: nitrogen-fixing gene *nifH*, B: ammonia-oxidizing archaea gene AOA, C: ammonia-oxidizing bacteria AOB, D: denitrifying bacteria *nirK*.

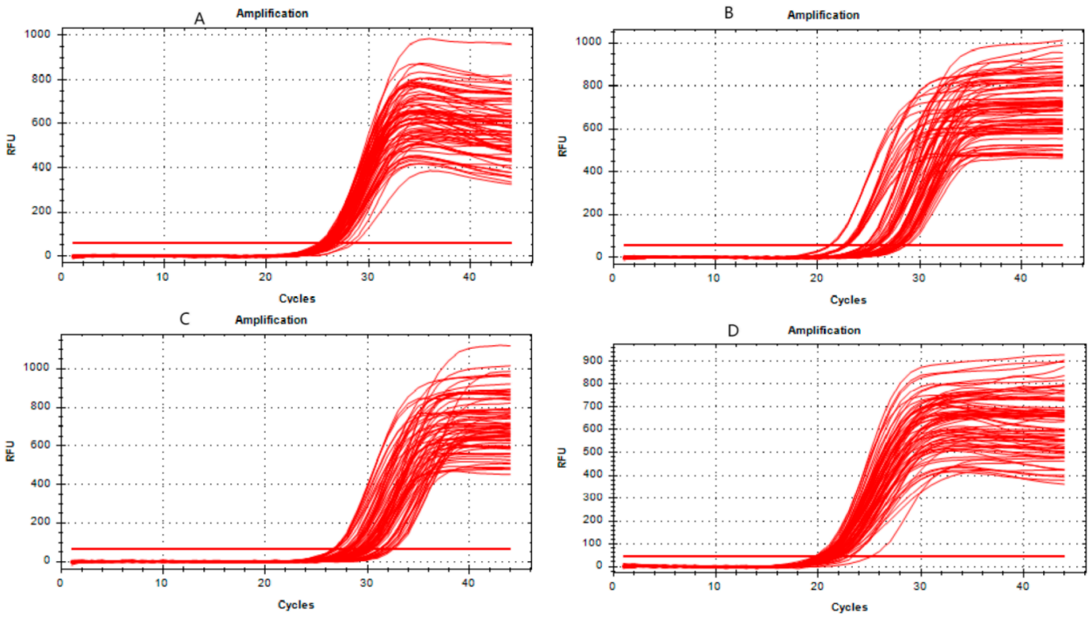

Figure S4 Amplification curve of nitrogen cycle functional genes by real-time flourescence quantitative PCR

Footnote: A: nitrogen-fixing gene *nifH*, B: ammonia-oxidizing archaea gene AOA, C: ammonia-oxidizing bacteria AOB, D: denitrifying bacteria *nirK*.

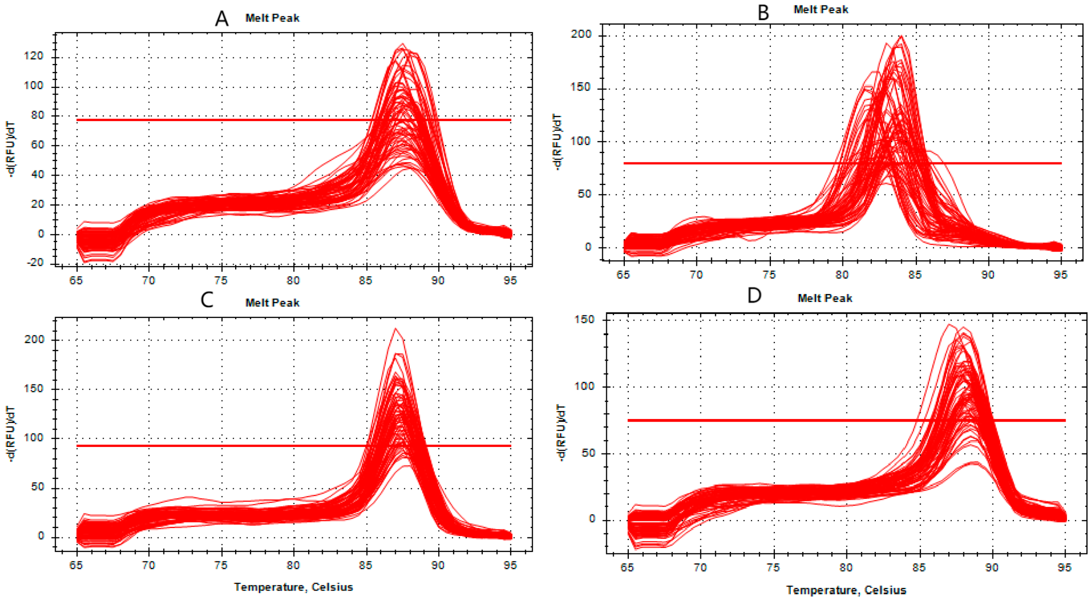

Figure S5 Melting curve of nitrogen cycle functional genes by real-time flourescence quantitative PCR

Footnote: A: nitrogen-fixing gene *nifH*, B: ammonia-oxidizing archaea gene AOA, C: ammonia-oxidizing bacteria AOB, D: denitrifying bacteria *nirK*.

Table S3 Effects and interactions of plant species, planting patterns and nitrogen addition on plant height (cm· plant^{-1}) and biomass (g· plant^{-1})

Factor	Height	Total biomass
Flora	7369.415**	5438.231**
Cropping pattern	120.875**	45.694**
Nitrogen addition	71.702**	43.304**
Flora×Cropping pattern	1.562	62.261**
Flora×Nitrogen addition	89.183**	39.878**
Cropping pattern×Nitrogen addition	5.141**	4.183**
Flora×Cropping Pattern×Nitrogen addition	7.226**	4.594**

Footnote:: "**" represents a very significant association ($P < 0.01$), and the number represents an F value.

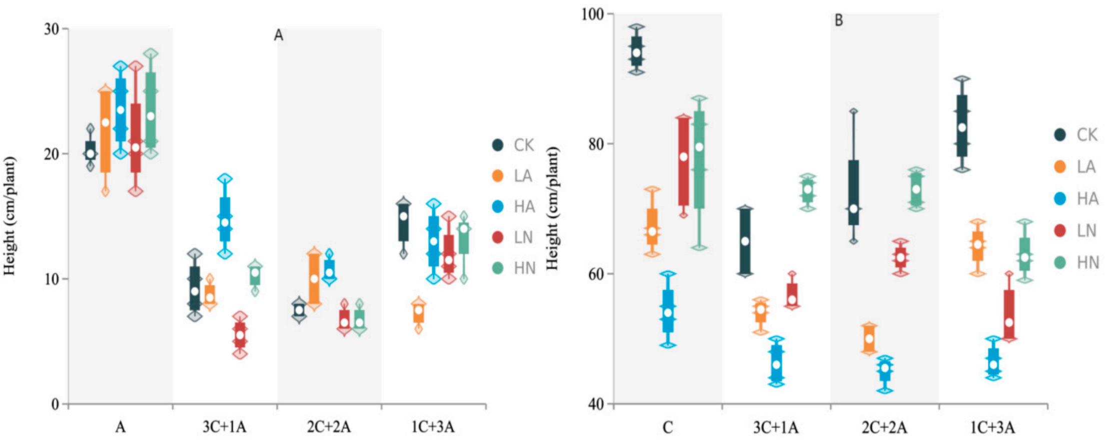

Figure S6 Plant height (A: Plant height of *A. cristatum* B: plant height of *C. spinifex*)

Footnote: A: Plant height of *A. cristatum*, C: *C. spinifex* single species, 3C+1A: *C. spinifex* and *A. cristatum* in a 3:1 ratio, 2C+2A: *C. spinifex* and *A. cristatum* in a 2:2 ratio, 1C+3A: *C. spinifex* and *A. cristatum* in a 1:3 ratio. CK: control, LA: low concentration ammonium nitrogen, HA: high concentration ammonium nitrogen, LN: low concentration nitrate nitrogen, HN: high concentration nitrate nitrogen. Different capital letters indicate that under the same nitrogen addition level, there are significant differences among different planting patterns ($P < 0.05$). Under the same planting pattern with different lowercase letters, there were significant differences among different nitrogen supplemental levels ($P < 0.05$).

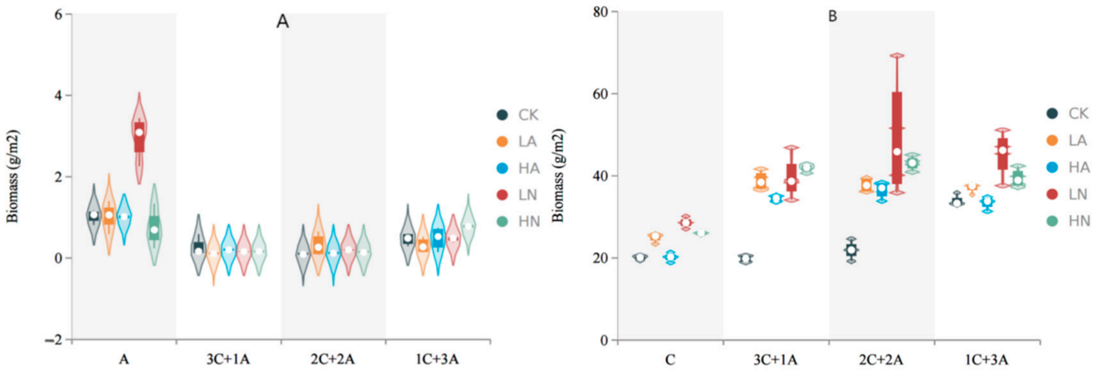

Figure S7 Plant biomass (A: total biomass of *A. cristatum* B: total biomass of *C. spinifex*)

Footnote: A: Plant height of *A. cristatum*, C: *C. spinifex* single species, 3C+1A: *C. spinifex* and *A. cristatum* in a 3:1 ratio, 2C+2A: *C. spinifex* and *A. cristatum* in a 2:2 ratio, 1C+3A: *C. spinifex* and *A. cristatum* in a 1:3 ratio. CK: control, LA: low concentration ammonium nitrogen, HA: high concentration ammonium nitrogen, LN: low concentration nitrate nitrogen, HN: high concentration nitrate nitrogen. Different capital letters indicate that under the same nitrogen addition level, there are significant differences among different planting patterns ($P < 0.05$). Under the same planting pattern with different lowercase letters, there were significant differences among different nitrogen supplemental levels ($P < 0.05$).

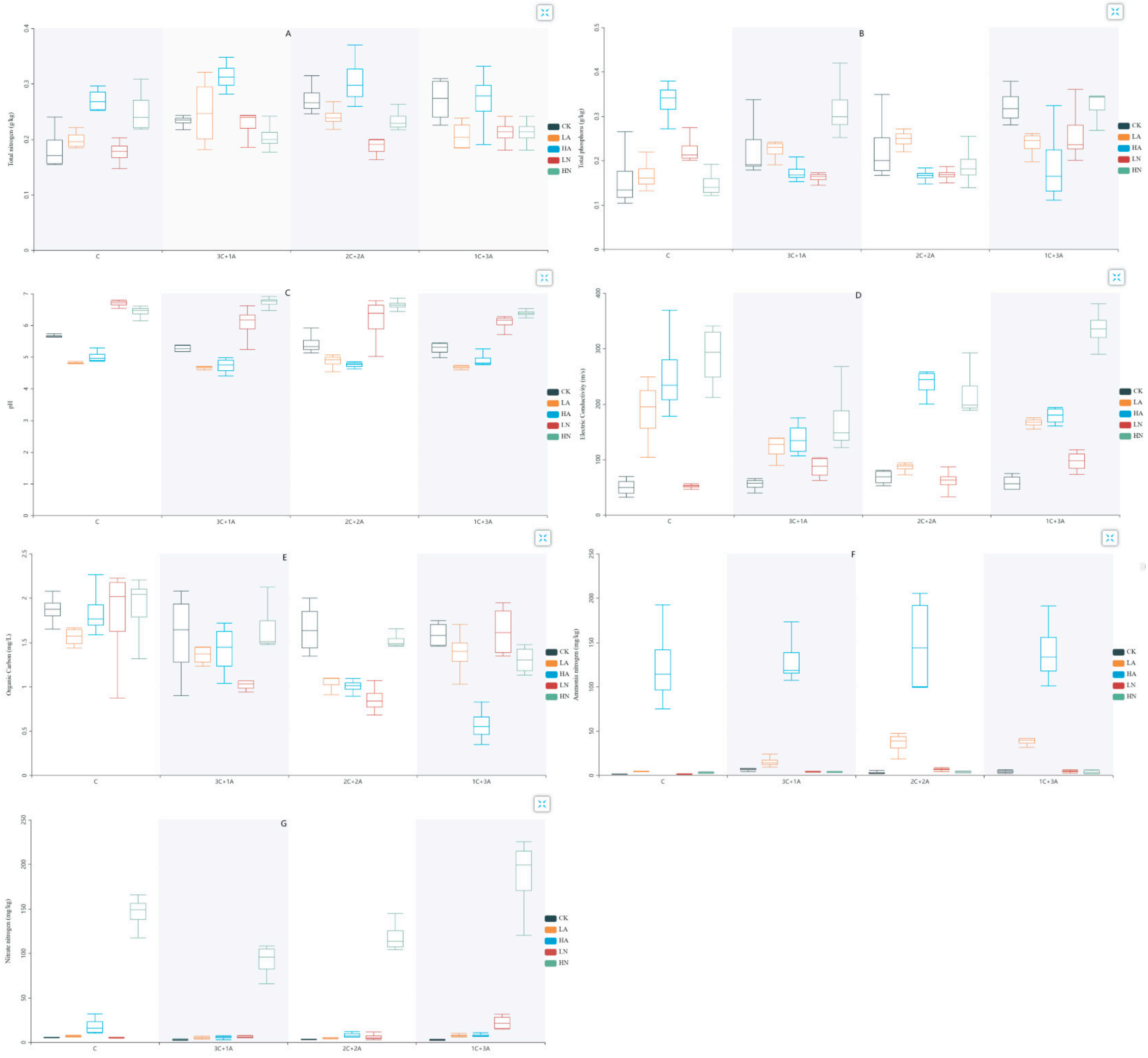

Figure S8 Physical and chemical properties of rhizosphere soil (A: TN, B: TP, C: soil pH, D: EC, E: SOC, F: NH_4^+-N, G: NO_3^+-N)

Footnote: A: Plant height of *A. cristatum*, C: *C. spinifex* single species, 3C+1A: *C. spinifex* and *A. cristatum* in a 3:1 ratio, 2C+2A: *C. spinifex* and *A. cristatum* in a 2:2 ratio, 1C+3A: *C. spinifex* and *A. cristatum* in a 1:3 ratio. CK: control, LA: low concentration ammonium nitrogen, HA: high concentration ammonium nitrogen, LN: low concentration nitrate nitrogen, HN: high concentration nitrate nitrogen. Different capital letters indicate that under the same nitrogen addition level, there are significant differences among different planting patterns ($P < 0.05$). Under the same planting pattern

with different lowercase letters, there were significant differences among different nitrogen supplemental levels ($P < 0.05$).

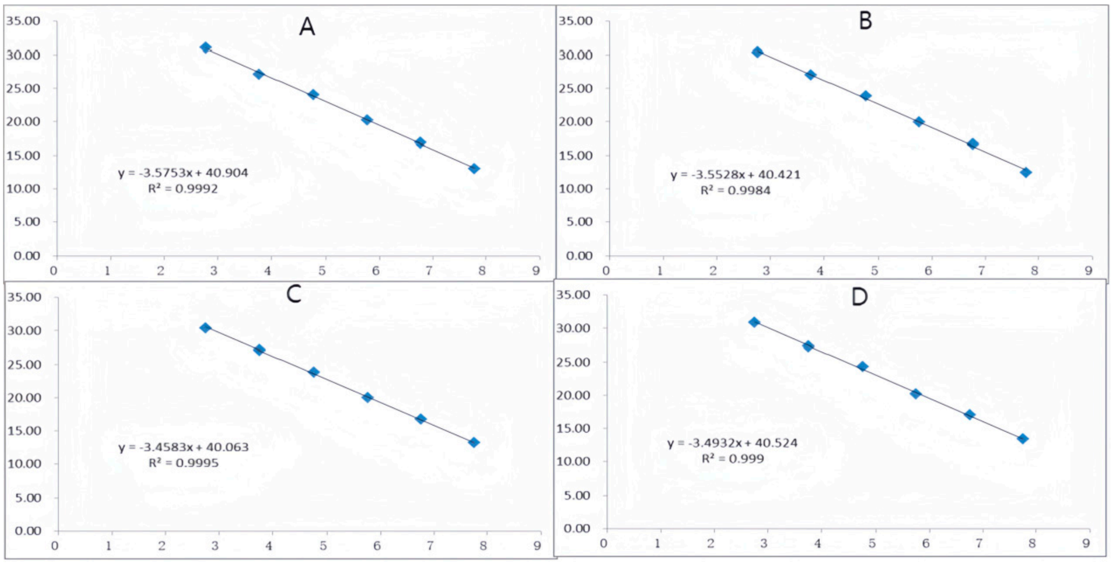

Figure S9 Standard curve of nitrogen cycle functional genes by real-time flourescence quantitative PCR

Footnote: A: nitrogen-fixing gene *nifH*, B: ammonia-oxidizing archaea gene AOA, C: ammonia-oxidizing bacteria AOB, D: denitrifying bacteria *nirK*.

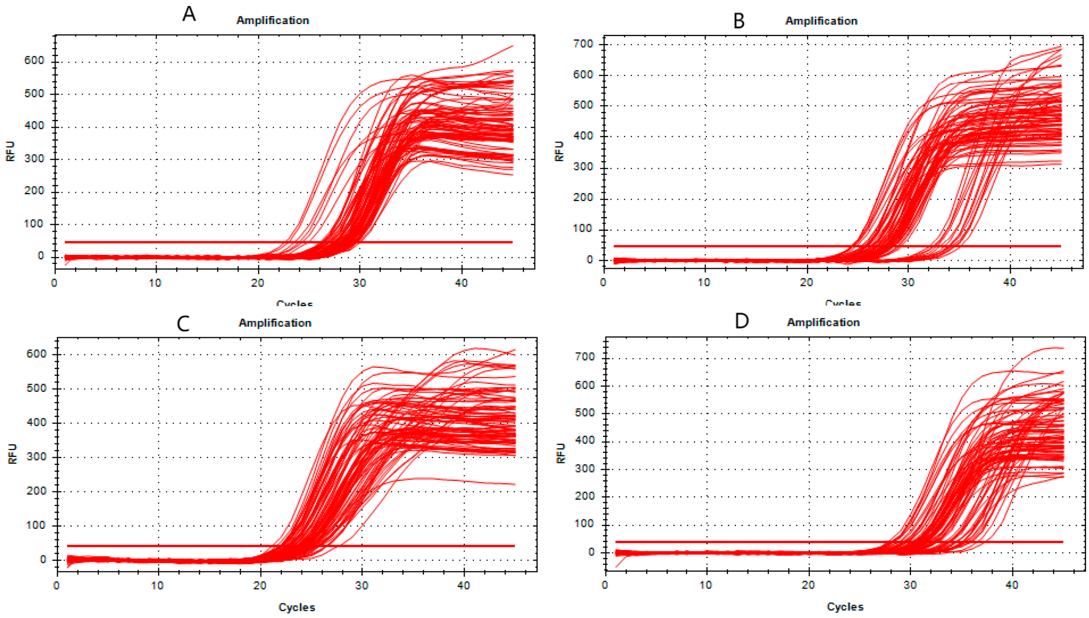

Figure S10 Amplification curve of nitrogen cycle functional genes by real-time flourescence quantitative PCR

Footnote: A: nitrogen-fixing gene *nifH*, B: ammonia-oxidizing archaea gene AOA, C: ammonia-oxidizing bacteria AOB, D: denitrifying bacteria *nirK*.

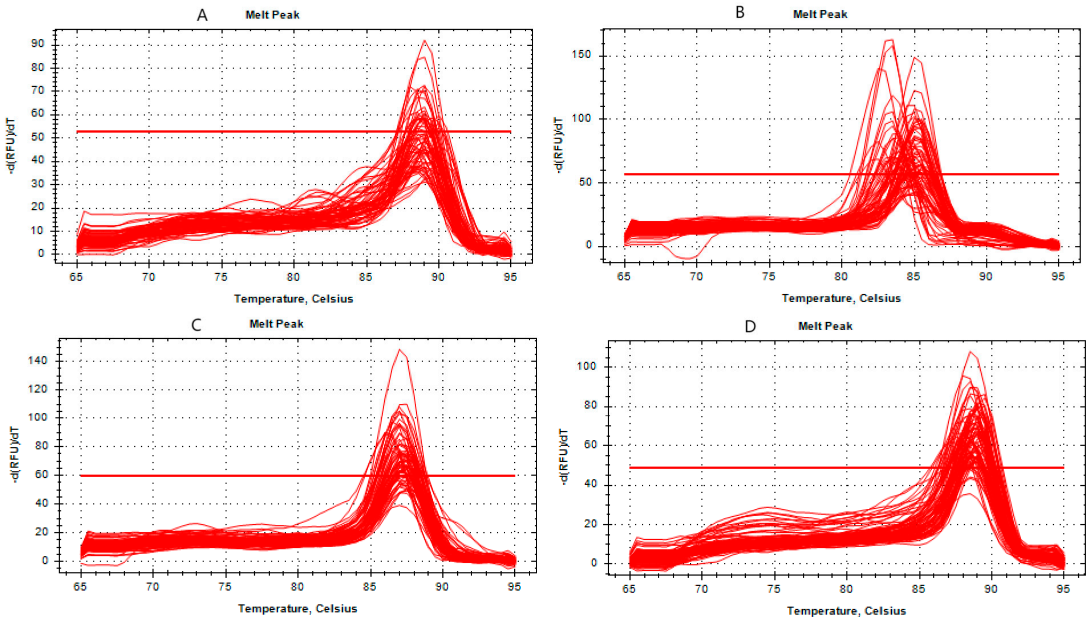

Figure S11 Melting curve of nitrogen cycle functional genes by real-time flourescence quantitative PCR

Footnote: A: nitrogen-fixing gene *nifH*, B: ammonia-oxidizing archaea gene AOA, C: ammonia-oxidizing bacteria AOB, D: denitrifying bacteria *nirK*.
